# LysM Proteins Regulate Fungal Development and Contribute to Hyphal Protection and Biocontrol Traits in *Clonostachys rosea*

**DOI:** 10.3389/fmicb.2020.00679

**Published:** 2020-04-16

**Authors:** Mukesh Dubey, Heriberto Vélëz, Martin Broberg, Dan Funck Jensen, Magnus Karlsson

**Affiliations:** Department of Forest Mycology and Plant Pathology, Uppsala Biocenter, Swedish University of Agricultural Sciences, Uppsala, Sweden

**Keywords:** antagonism, biocontrol, biological control, *Clonostachys rosea*, fungal–fungal interactions, LysM effector, LysM protein, mycoparasitism

## Abstract

Lysin motif (LysM) modules are approximately 50 amino acids long and bind to peptidoglycan, chitin and its derivatives. Certain LysM proteins in plant pathogenic and entomopathogenic fungi are shown to scavenge chitin oligosaccharides and thereby dampen host defense reactions. Other LysM proteins can protect the fungal cell wall against hydrolytic enzymes. In this study, we investigated the biological function of LysM proteins in the mycoparasitic fungus *Clonostachys rosea*. The *C*. *rosea* genome contained three genes coding for LysM-containing proteins and gene expression analysis revealed that *lysm1* and *lysm2* were induced during mycoparasitic interaction with *Fusarium graminearum* and during colonization of wheat roots. *Lysm1* was suppressed in germinating conidia, while *lysm2* was induced during growth in chitin or peptidoglycan-containing medium. Deletion of *lysm1* and *lysm2* resulted in mutants with increased levels of conidiation and conidial germination, but reduced ability to control plant diseases caused by *F*. *graminearum* and *Botrytis cinerea*. The Δ*lysm2* strain showed a distinct, accelerated mycelial disintegration phenotype accompanied by reduced biomass production and hyphal protection against hydrolytic enzymes including chitinases, suggesting a role of LYSM2 in hyphal protection against chitinases. The Δ*lysm2* and Δ*lysm1*Δ*lysm2* strains displayed reduced ability to colonize wheat roots, while only Δ*lysm1*Δ*lysm2* failed to suppress expression of the wheat defense response genes *PR1* and *PR4*. Based on our data, we propose a role of LYSM1 as a regulator of fungal development and of LYSM2 in cell wall protection against endogenous hydrolytic enzymes, while both are required to suppress plant defense responses. Our findings expand the understanding of the role of LysM proteins in fungal-fungal interactions and biocontrol.

## Introduction

Lysin motif (LysM) domains are approximately 50 amino acids long family 50 carbohydrate-binding modules (CBM50) that bind to *N*-acetylglucosamine (GlcNAc)-containing glycans such as peptidoglycan, chitin and chitin-like compounds (Buist et al., 2008). LysM-containing proteins can comprise one or multiple LysM modules and show high amino acid sequence variability (Buist et al., 2008; Akcapinar et al., 2015). LysM modules were first discovered in the antimicrobial enzyme lysozyme of a bacteriophage (Garvey et al., 1986), and subsequently identified in proteins from all kingdoms of life including bacteria, fungi, plants and animals (Buist et al., 2008; Kombrink and Thomma, 2013; Akcapinar et al., 2015). In bacteria, LysM modules are found in various proteins including hydrolytic enzymes like peptidoglycan hydrolases, peptidases, chitinases, esterases and nucleotidases, as well as in adhesins required for cell growth and division, autolysis, and virulence (Buist et al., 2008). In plants, LysMs are present in certain cell surface receptor kinases and recognize chitin, peptidoglycan and their derivatives (Antolín-Llovera et al., 2014), which are essential structural components of the fungal and bacterial cell wall, respectively. Also, they are a well-established microbe-associated molecular pattern (MAMP) in fungi and bacteria. LysM modules are also present in plants’ Nod factor receptors and Myc factor receptors that bind Nod factors from symbiotic rhizobia and Myc factors from symbiotic arbuscular mycorrhizal fungi, respectively. Detection of these compounds by transmembrane LysM-containing protein receptors of host plants activate MAMP-triggered defense signaling, against bacterial and fungal pathogens, and symbiotic signaling during interactions between legume plants and nitrogen-fixing bacteria and arbuscular mycorrhiza (Antolín-Llovera et al., 2014).

In fungi, LysM modules can be found either as part of secreted proteins with varying number of LysMs, but without any known catalytic domain (referred to as LysM effectors), or together with known catalytic protein modules (referred to as LysM-containing proteins) (de Jonge and Thomma, 2009; Martinez et al., 2012). Examples of LysM-containing proteins include glycoside hydrolases (chitinases), CyanoVirin-N homology (CVNH) modules, *N*-acetylmuramoyl-L-alanine amidase modules and polysaccharide deacetylase type 1 modules (de Jonge and Thomma, 2009). Based on evolutionary analyses, the number and consensus pattern of cysteine (Cys) residues in the LysM module divides them into a fungal-specific group and a fungal/bacterial group, respectively (Akcapinar et al., 2015; Cen et al., 2017; Kombrink et al., 2017). The fungal-specific group contains three conserved Cys residues in the LysM module and one Cys residue outside of the module, which putatively forms two disulfide bridges. In contrast, the fungal/bacterial group contains no or only one Cys residue at a different position than those found in fungal-specific LysMs (Akcapinar et al., 2015).

Functional characterization of LysM proteins from fungal plant pathogens revealed that LysM effectors can suppress chitin-triggered immunity in host plants by sequestering the chitin oligosaccharides released from the fungal cell walls, and thereby contribute to fungal virulence (de Jonge et al., 2010; Marshall et al., 2011; Mentlak et al., 2012; Takahara et al., 2016; Cen et al., 2017; Kombrink et al., 2017). Furthermore, LysM effectors Mg3LysM and Vd2LysM (from the septoria tritici blotch fungus, *Zymoseptoria tritici*, and the soil-borne vascular wilt pathogen, *Verticillium dahliae*, respectively), are shown to protect fungal hyphae against the hydrolytic activity of host chitinase (Marshall et al., 2011; Cen et al., 2017; Kombrink et al., 2017). In contrast to these findings, deletion of four LysM effector genes that are highly expressed in the blue mold fungus, *Penicillium expansum*, during the infection of apple, and three core LysM effector genes (*Vd4LysM*, *Vd5LysM* and *Vd6LysM*) conserved in several *V. dahliae* strains, did not affect their virulence on their host plants (Kombrink et al., 2017; Levin et al., 2017). The biological role of LysM effectors has also been investigated in the insect-pathogenic fungus, *Beauveria bassiana*. Analysis of gene deletion mutants showed that the LysM effector Blys2 and Blys5 are necessary for the full virulence of *B*. *bassiana* against its insect hosts. However, only Blys5 could protect fungal hyphae against chitinase hydrolysis (Cen et al., 2017).

Interestingly, these characterized LysM proteins belong mainly to the fungal/bacterial group and are LysM effectors (de Jonge and Thomma, 2009; Akcapinar et al., 2015). The vast majority of genes encoding LysM effectors and LysM-containing proteins in fungal species with distinct lifestyles, including plant pathogens, saprotrophs and mycotrophs (de Jonge and Thomma, 2009; Gruber et al., 2011; Martinez et al., 2012; Kombrink et al., 2017), remain uncharacterized. Therefore, more functional studies are required to understand (i) whether there is a distinction in function between fungal-specific and fungal/bacterial LysM proteins, and (ii) the role of LysM proteins in filamentous fungi, including those with saprotrophic and mycotrophic life style. Considering the fact that LysM proteins show high diversity in protein length, LysM module number, and amino acid sequence between closely related fungal species and even between allelic proteins (de Jonge and Thomma, 2009; Akcapinar et al., 2015; Cen et al., 2017), it is utmost important to characterize their functions in fungi with diverse life styles in order to gain insights into the range of biological roles of LysM proteins. For example, external application of purified TAL6, which is a fungal-specific LysM effector from the mycoparasitic fungus *Trichoderma atroviride*, inhibited spore germination of *Trichoderma* spp. but not of other fungi, suggesting a species-specific role of fungal LysM modules in regulation of fungal growth and development (Seidl-Seiboth et al., 2013). In addition, *Tal6* deletion strains showed reduced mycoparasitic ability toward the host fungus (Romero-Contreras et al., 2019). The results from these studies highlight a possible difference in function between fungal-specific and fungal/bacterial LysM effectors (Seidl-Seiboth et al., 2013).

The filamentous fungus *Clonostachys rosea* (Link: Fr.) Schroers, Samuels, Seifert & W. Gams, comb. nov. (Schroers et al., 1999) has a complex life style as a necrotrophic mycoparasite by killing other fungi and feeding on dead mycelia, as a saprotroph by digesting organic material in soil and as a beneficial plant root colonizer (Sutton et al., 1997; Ravnskov et al., 2006; Karlsson et al., 2015). *C*. *rosea* strain IK726 is known to be an effective biological control agent against a broad range of fungal plant pathogens and plant parasitic nematodes (Jensen et al., 2000, 2004, 2016; Cota et al., 2008; Iqbal et al., 2018). The aim of the present study was to investigate the biological role of fungal-specific LysM proteins in *C. rosea*, with emphasis on their function in mycoparasitic fungal-fungal and beneficial fungal-plant interactions. By generating and analyzing gene deletion and complementation strains, we demonstrate a role of LysM proteins in regulating fungal development and autolysis, in biological control of plant pathogens, in suppression of plant host defense response and in root colonization. Our findings expand the understanding of the role of LysM proteins in biological interactions between filamentous fungi and with plants.

## Materials and Methods

### Fungal Strains and Culture Conditions

*Clonostachys rosea* strain IK726 wild type (WT; Knudsen et al., 1995) and mutants derived from it, *Botrytis cinerea* strain B05.10 (Amselem et al., 2011) and *Fusarium graminearum* strain 1104-14 (Fusarium collection at the National Veterinary Institute in Norway) were maintained on potato dextrose agar (PDA; Oxoid, Cambridge, United Kingdom) medium at 25°C. SMS medium (Dubey et al., 2012) supplemented with 1% glucose or Czapek-dox (CZ) was used to grow *C*. *rosea* for gene expression analysis unless otherwise specified.

### Gene Identification and Sequence Analysis

The *C*. *rosea* strain IK726 genome version 1 (Karlsson et al., 2015) and version 2 (Broberg et al., 2018), were screened for the presence of genes encoding LysM-containing protein by BLASTP analysis using amino acid sequences of LysM containing proteins from a diverse group of fungi (Supplementary Table S1). Presence of conserved domains were analyzed with Simple modular architecture research tool (SMART) (Bork et al., 2009), InterProScan (Quevillon et al., 2005) and conserved domain search (CDS) (Marchler-Bauer et al., 2011). Presence of Cys residues in specific spacing pattern was analyzed manually. Signal P 4.1 (Petersen et al., 2011) was used to search for signal peptide cleavage sites. Amino acid sequence alignment was performed using ClustalW2 (Larkin et al., 2007) with default settings for multiple sequence alignment. Single nucleotide polymorphisms (SNPs) in *lysm1*, *lysm2* and *chiC2* were detected using a procedure described previously (Broberg et al., 2018). A multidimensional scaling (MDS) was performed using R 3.5 on SNPs occurrence in *lysm1*, *lysm2* and *chiC2* to visualize the sequence variation across isolates.

### Phylogenetic Analysis and Molecular Evolution

LysM module protein sequences were aligned using Muscle (Edgar, 2004) and phylogenetic analysis was performed using maximum likelihood methods implemented in MEGA ver. 6 (Tamura et al., 2013). The WAG (Whelan and Goldman) with G (Gamma distribution) amino acid substitution model (Whelan and Goldman, 2001) was used with pairwise deletion of gaps. Statistical support for branches was assessed by 1000-iteration bootstrap resampling. Recombination breakpoints were identified using the program GARD (Kosakovsky-Pond et al., 2006) implemented in the HyPhy software package (Kosakovsky-Pond et al., 2005), through the Datamonkey webserver (Kosakovsky-Pond et al., 2005). The rate of non-synonymous and synonymous substitutions at each codon, and identification of sites evolving under positive or negative selection, was determined using the random effects maximum likelihood models (REL) using default settings (Kosakovsky-Pond and Frost, 2005a, b). A Bayes factor value ≥ 10 was used as an indication of positive selection at a site.

### Gene Expression Analysis

Gene expression analyses of *lysm1* and *lysm2* were carried out in *C*. *rosea* strain IK726 under the following conditions: (1) during dual plate interaction with the fungal preys, *B. cinerea* or *F. graminearum*, (2) during interaction with wheat roots, (3) in culture filtrates from *F*. *graminearum*, and *C*. *rosea* itself, (4) in liquid SMS amended with 1% colloidal chitin or 0.1% peptidoglycan, and (5) in germinated conidia in liquid SMS.

For LYSM1 and LYSM2 gene expression analysis during interactions, *C*. *rosea* was confronted with *B*. *cinerea* (Cr-Bc) or *F*. *graminearum* (Cr-Fg) on agar plates as described previously (Dubey et al., 2014b, 2016). The growing front (7–10 mm) of *C*. *rosea* was harvested at mycelial contact (C; early physical contact between the mycelia) and at 24 h post mycelial contact (AC; close physical contact). *C*. *rosea* confronted with itself (Cr-Cr) was used as control treatment.

For expression analyses of *lysm1* and *lysm2* in *C*. *rosea* during the interaction with wheat roots (Cr-Wr), wheat seeds were surface sterilized with 2% sodium hypochlorite (VWR international, Fontenay-sous-Bois, France) for 10 min under constant shaking condition on a rotary shaker at 100 rpm. The seeds were then washed several times with sterile water to remove traces of chlorine. Surface sterilized seeds were germinated on sterilized moist filter paper placed in 9-cm petri plates (five seeds per plate) in dark at 20°C. Three days old wheat seedlings were inoculated by dipping the roots for 5 min in *C*. *rosea* spore suspensions (1e + 07 spore/ml) in sterile condition, transferred back to the filter paper in petri plates and incubated at 20°C in a growth cabinet with 12 h light (CLF plant climates, GmbH, Germany). Roots were harvested 48 h and 96 h post inoculations (hpi).

Gene expression analysis of *lysm1* and *lysm2* was performed in culture filtrates from *F*. *graminearum*, and from *C*. *rosea* itself to monitor the response to the exogenous (*F*. *graminearum*) and endogenous (*C*. *rosea*) secreted factors including chitinases. *C*. *rosea* mycelia was cultivated and harvested 2 hpi as described previously (Dubey et al., 2014b, 2016). In brief, an agar plug of *C*. *rosea* was pre-cultivated for 5 days in 100 ml flasks containing 20 ml liquid CZ medium on rotary shaker (200 rpm) at 25°C. The mycelia were harvested by filtering through Miracloth. The harvested mycelium was washed with sterile distilled water and then transferred to new flasks containing 20 ml culture filtrates of *C. rosea* or *F. graminearum*. Mycelia transferred in fresh CZ medium were used as control treatment. Culture filtrates were obtained by growing *C*. *rosea* or *F*. *graminearum* in 200 ml liquid CZ medium as described previously (Dubey et al., 2014b).

For gene expression analysis in medium containing chitin or peptidoglycan (Sigma-Aldrich, St. Louis, MO, United States), *C*. *rosea* was pre-cultivated for 5 days in SMS medium supplemented with 1% glucose. Mycelia were harvested 3 days post inoculation (dpi), washed with sterilized distilled water and transferred to new flasks containing 20 ml SMS medium where 1% glucose was substituted with 1% colloidal chitin or 0.1% peptidoglycan as a sole carbon source (Dubey et al., 2012). Colloidal chitin was prepared from crab-shell chitin (Sigma-Aldrich, St. Louis, MO, United States) as following the procedure described previously (Seidl et al., 2005). Mycelia transferred in fresh SMS medium with 1% glucose was used as control treatment. Fungal mycelia were collected 2 hpi and washed in distilled water.

For *lysm1* and *lysm2* gene expression analysis in *C*. *rosea* germinated conidia (GC), conidia were harvested in sterile water from the surface of 2 weeks old PDA plates and the concentration was determined under the microscope using a bright line hemocytometer (Sigma-Aldrich, St. Louis, MO, United States). A conidial suspension (1e+07) was inoculated in 50 ml SMS medium to germinate. Samples were harvested 24 hpi by centrifugation. Agar plugs from the growing mycelial front were inoculated in 50 ml SMS medium to grow for 24 h and used as control treatment to compare gene expression in germinating conidia to mycelia. Harvested samples were immediately frozen in liquid nitrogen and stored at −80°C.

Gene expression analysis of PR1 (GenBank accession number: AF384143.1) and PR4 (Wheatwin-1; GenBank accession number: CAA06856.1) gene was performed in wheat roots inoculated with *C*. *rosea* WT and *lysm* deletion strains. The *C*. *rosea* – wheat interaction experiment was set up as described above and roots were harvested 120 hpi.

RNA was extracted using the Qiagen RNeasy kit following the manufacturer’s protocol (Qiagen, Hilden, Germany) and reverse transcribed using the iScript cDNA synthesis kit (Bio-Rad, Hercules, CA, United States). Transcript level was quantified by quantitative reverse transcription PCR (RT-qPCR) using gene specific primer pairs (Supplementary Table S3) as described previously (Kamou et al., 2016). For *lysm1* and *lysm2* gene expression analysis in *C*. *rosea*, relative expression levels for the target gene in relation to actin (GenBank accession number: MT037018; Supplementary Table S3) and β-tubulin gene (GenBank accession number: AF358197.1; Mamarabadi et al., 2008) were calculated from threshold cycle (Ct) values using the 2^–ΔΔ*Ct*^ method (Livak and Schmittgen, 2001). For PR1 and PR4 gene expression analysis in wheat roots, gene specific primers described previously (Roberti et al., 2008; Marshall et al., 2011) were used and expression data were normalized to expression of the wheat β-tubulin gene (GenBank accession number: U76744.1). Gene expression analysis was carried out in three or five biological replicates, each based on two technical replicates.

### Construction of Deletion Vector, Transformation and Mutant Validation

Three fragment multisite gateway cloning system (Invitrogen, Carlsbad, CA, United States) was used to construct gene deletion vectors. The ∼ 1 kb 5′-flank and 3′-flank regions of *lysm1* and *lysm2* were amplified from genomic DNA of *C*. *rosea* using gene specific primer pairs 568 ups F/568 ups R and 568 ds F/568 ds R; and 6500 ups F/6500 ups R and 6500 ds F/6500ds R, respectively, as indicated in Supplementary Figure S3. Gateway entry clones of the purified 5′-flank and 3′-flank PCR fragments were generated as described by the manufacturer (Invitrogen, Carlsbad, CA, United States). The hygromycin resistance cassette (hygB) generated during our previous studies (Dubey et al., 2012, 2013a) from pCT74 vector, as well as a chlorimuron ethyl resistance cassette (ILV1) generated as a PCR product from the pCB1532 vector (Sweigard et al., 1997), were used. The gateway LR recombination reaction was performed using entry plasmid of respective fragments and destination vector pPm43GW (Karimi et al., 2005) to generate the deletion vectors.

Complementation cassettes for *lysm1* and *lysm2* were constructed by PCR amplification of the full-length sequence of *lysm1* and *lysm2* including more than 1 kb upstream and around 500 bp downstream regions from genomic DNA of *C. rosea* WT using 568 ups F/568 comp R and 6500 ups F/6500 comp R primers, respectively (Supplementary Table S3). The amplified DNA fragments were purified and integrated into destination vector pPm43GW as described above using Gateway cloning technology to generate complementation vectors.

*Agrobacterium tumefaciens*-mediated transformation (ATMT) was performed based on a previous protocol for *C. rosea* (Utermark and Karlovsky, 2008). Transformed strains were selected on plates containing either hygromycin for single gene deletion; chlorimuron ethyl for complementation; and both antibiotics, in the case of double gene deletion. Validation of homologous integration of the deletion cassettes in putative transformants were performed using a PCR screening approach with primer combinations targeting the hygB cassette (i.e., for *lysm1* or *lysm2* deletions) or ILV1 cassette (i.e., for *lysm1lysm2* double-deletions) and sequences flanking the deletion cassettes (Supplementary Figure S3), as described previously (Dubey et al., 2013a, b). The PCR positive transformants were tested for mitotic stability, and were purified by two rounds of single spore isolation (Dubey et al., 2012). Transcript level of *lysm1* and *lysm2* on WT, and respective gene deletion and complementation strains, was determined by RT-PCR using RevertAid premium reverse transcriptase (Fermentas, St. Leon-Rot, Germany) and their respective primer pairs (Supplementary Table S3).

Phenotypic analyses experiments were performed with *C*. *rosea* WT, single deletion strain *lysm1* (Δ*lysm1*) and *lysm2* (Δ*lysm2*) and their respective complemented strains Δ*lysm1*+ and Δ*lysm2*+, and two *lysm1lysm2* double deletion strains (Δ*lysm1*Δ*lysm2*A and Δ*lysm1*Δ*lysm2*B). Each experiment included three to five biological replicates (depending on the phenotype), and each experiment was repeated two times with similar results unless otherwise specified.

### Phenotypic Analyses

To analyze the growth rate and colony morphology, a three mm agar plug from the growing mycelial front was transferred to solid PDA or CZ and colony diameter was measured after 5 days of growth at 25°C. Colony morphology was observed weekly, photographs were taken by scanning the Petri dishes using Epson Perfection V700 Photo scanner (Epson, Suwa, Japan), and pixel intensity was analyzed with the ImageJ image analysis software (Schneider et al., 2012). Pixel intensity values from the colony morphology of the *lysm* deletion and complementation strains were normalized to the values of the WT and were used as proxy for mycelial disintegration and biomass measurement.

Conidial yield and germination was determined following the procedure described before (Fatema et al., 2018). For conidiation analysis, conidia were harvested from a 12-day-old plate in 10 ml distilled water, and filtered through Miracloth to remove the mycelial debris. Conidial concentration was determined under the microscope using a bright line hemocytometer (Sigma-Aldrich, St. Louis, MO, United States).

For conidial germination, a conidial suspension (1e+05) was placed on microscope slides with half-strength PDA. The slides were then transferred to nine-cm plates, covered with the lid and incubated at 25°C in dark. After 8 and 24 h of incubation, photographs of the germinating conidia were recorded in a Leica DM5500M Microscope at 20 X magnification equipped with a Leica DFC360FX digital camera (Wetzlar, Germany). The experiment was performed in four biological replicates. For conidia germination, two hundred conidia were counted while 50–100 conidia were used for germ tube measurement for each replicate. The growth rate and conidiation experiment was performed in four biological replicates.

Antagonistic behavior against the phytopathogenic fungi, *B*. *cinerea* and *F. graminearum*, was tested using an *in vitro* plate confrontation assay on PDA medium as described before (Dubey et al., 2014a, b, 2016). The growth of *B*. *cinerea* and *F. graminearum* was measured daily until their mycelial front touched the *C*. *rosea* mycelial front, while the growth of *C. rosea* strains was measured until the fungus reached another side in the plate. Plates were scanned and the photographs were used to measure the mycelial biomass as described above.

To test the hyphal protection of *C. rosea* WT, deletion and complemented strains against secreted factors including the hydrolytic enzymes from *B*. *cinerea* or *F. graminearum*, mycelial biomass production of *C*. *rosea* WT and deletion strains was measured in culture filtrates from *B*. *cinerea* or *F. graminearum*. The mycelial biomass was measured following procedure described previously (Dubey et al., 2014a, b, 2016). The plate confrontation assay and culture filtrates test was performed in five biological replicates.

### Protoplast Formation Assay

Protoplasts formation was done as previously described by Sun et al. (2017), with some modifications. Spores (1e+06) of *C. rosea* WT and *lysm* deletion strains were incubated in 250-mL flasks containing 20 mL of potato dextrose broth (PDB) and placed at 26°C for 2 days. The culture was mixed 1:1 with 1.4 M NaCl solution containing 80 mg mL^–1^ of snailase enzyme mix (Bio Basic Inc., Markham, Ontario Canada) to hydrolyze the mycelia. Chitinase activity of snailase enzyme mix has been shown previously (Boller et al., 1983; Xiao et al., 2007). The cultures were placed in a water bath at 28°C for 3 h. Protoplast release was monitored by removing 200 μL aliquots every 30 min and counting using a hemocytometer (Sigma-Aldrich, St. Louis, MO, United States). The experiment was performed in five replicates.

### Enzyme Activity Assays

To measure chitinase activity, conidial suspensions (1e+07) were inoculated into 100 ml flasks containing 50 ml of liquid SMS with 1% glucose and were shaken on rotary shaker (150 rpm) at 25°C. Chitinase activity was measured using (GlcNAc)_2_ conjugated to 4-methylumbelliferyl (4-MU) as substrates (Sigma-Aldrich, St. Louis, MO, United States) as described previously (Dubey et al., 2012). Fluorescence of released 4-MU was determined by using a luminescence spectrometer model Fluostar Omega (BMG Labtech, Germany) at wave length Ex350/Em455 and gain 1500. The experiment was performed in five biological replicates and each replicate consisted of two technical replicates.

### *In planta* Biocontrol Assay Against *B. cinerea* and *F*. *graminearum*

The biocontrol ability of *C. rosea* strains against *B. cinerea* and *F*. *graminearum* was evaluated in an *Arabidopsis thaliana* detached-leaf and fusarium foot rot assay as described previously (Knudsen et al., 1995). Briefly, detached leaves of *A. thaliana* ecotype Columbia-0 (Col-0) were inoculated with a 5-μl droplet of conidial suspension (1e+06 conidia/ml) derived from *C. rosea* strains and allowed to dry for 60 min. Then, the same spot was re-inoculated with a 5-μl conidial suspension (1e+06 conidia/ml) of *B. cinerea*. The diameter of necrotic lesions was measured 60 h post inoculation as described previously (Dubey et al., 2014a, 2016). The experiments were performed in five biological replicates and each replicate consisted of six leaves for each treatment.

For fusarium foot rot assay, surface sterilized wheat seeds were treated with *C. rosea* conidia (1e+07 conidia/ml) in sterile water, sown in moistened sand, and kept in a growth chamber after pathogen inoculation (Dubey et al., 2014b, 2016). Plants were harvested 3 weeks post inoculation and disease symptoms were scored on 0-4 scale as described before (Knudsen et al., 1995; Dubey et al., 2014b, 2016). The experiment was performed in five biological replicates with 15 plants in each replicate.

### Root Colonization

Wheat seeds were surface sterilized and germinated as described in the gene expression section. The roots were then inoculated with conidial suspension of *C*. *rosea* strains (1e+07 conidia/ml) as described in gene expression section and were co-cultivated for 5 days at room temperature. For each treatment five biological replicates with five seedlings per replicate were used. To quantify the root colonization, the roots were detached from the plant five dpi, washed with water and used for DNA extraction. The concentration of DNA was determined spectrophotometrically using a nanodrop (Thermo Fisher Scientific, Wilmington, DE, United States) and 100 ng was used to quantify the DNA level of *C*. *rosea* strains in wheat roots using qPCR (Dubey et al., 2013a). The actin gene was used as target gene for *C*. *rosea*, and *Hor1* (Nicolaisen et al., 2009) was used as target gene for wheat. Root colonization was expressed as the ratio between *actin* and *Hor1*.

To quantify the internal root colonization, water-washed roots were surface sterilized with 2% sodium hypochlorite (VWR international, Fontenay-sous-Bois, France) for 2 min and washed two times with sterilized distilled water to remove the traces of sodium hypochlorite. The optimum concentration of sodium hypochlorite was selected based on successive screening of *C*. *rosea* to this compounds (Dubey et al., 2014a). The roots were blot dried, weighed, and homogenized in two ml phosphate buffer pH 7.4 with 0.01% Triton X-100 using a RETSCH MM 400 Mixer Mill (Thermo Fisher Scientific, Wilmington, DE, United States) with 20 HZ for 15 s. Serial dilutions were plated on PDA plates containing Rose Bengal 50 μg/ml, chloramphenicol 25 μg/ml and kanamycin 50 μg/ml (Sigma-Aldrich, St. Louis, MO, United States) to count the colony forming units (cfus).

### Statistical Analysis

Analysis of variance (ANOVA) was performed on gene expression and phenotype data using a General Linear Model approach implemented in Statistica version 13 (TIBCO Software Inc., Palo Alto, CA, United States). Pairwise comparisons were made using the Fisher’s or Tukey–Kramer method at the 95% significance level. Student’s *t*-test was also performed on gene expression data.

## Results

### Identification and Sequence Analysis of Predicted LysM Proteins

Analysis of the *C*. *rosea* strain IK726 genome version 1 (Karlsson et al., 2015) and version 2 (Broberg et al., 2018) using 35 protein sequences with variable number (1 – 7) of LysM modules and one protein sequences with the additional chitin binding module (Supplementary Table S1) from taxonomically different fungal species, identified three genes predicted to encode proteins containing LysM modules. One gene (protein ID: CRV2G00000260) was already identified as *chiC2* (GenBank accession number: MT037003), encoding a killer toxin-like chitinase (Tzelepis et al., 2015). The second gene model predicted to encode protein ID CRV2G00003915 was named *lysm1* (GenBank accession number: MT037001). Comparisons between the *C. rosea* ver. 1 and ver. 2 gene models of the third gene revealed a C-terminal abhydrolase module present in ver. 2 but not in ver. 1. RNA-seq read coverage from Karlsson et al. (2015) showed that the ver. 1 gene model (protein ID: BN869_T00006500) was correct and this was named *lysm2* (GenBank accession number: MT037002). The characteristics of LYSM1, LYSM2 and CHIC2 are presented in Table 1.

**TABLE 1:** Characteristics of the putative LysM proteins in *Clonostachys rosea*, *C*. *chloroleuca*, and *C*. *solani*.

Name/	Signal peptide/	Genomic	Coding	No of	Protein	LysM	No. Cys
protein ID	Transmembrane^§^	sequences	sequences	introns	length	modules^§^	residues^*λ̄*^
LYSM1^#^	Transmembrane (87–109)	534 bp	531 bp	0	177 aa	1 (127–171)	4 (3)
LYSM2	Signal peptide (1–23)	1513 bp	1392 bp	2	464 aa	2 (235–280; 289–339)	22 (3; 3)
CHIC2	Signal peptide (1–20)	2955 bp	2598 bp	2	866 aa	2 (309–354; 372–419)	32 (3; 5)
MT037016^#^	No	1440	1341	1	447 aa	1 (121–165)	7 (1)
MT037017^#^	No	852	828	1	276 aa	3 (5–47; 122–160; 229–275)	11 (3; 3; 3)

*ChtBD1, chitin-binding (CBM18) module; GH18, glycoside hydrolase family 18 module. ^#^Secreted through a non-classical secretion pathway (SecretomeP score ≥ 0.91). ^§^Number in parenthesis denotes the amino acid positions from N-terminus. CHIC2 contains additional modules ChtBD1 and GH18 between the amino acid position 443–449 and 513–862, respectively. ^*λ̄*^Number in parenthesis denotes the Cys residues present in each LysM module. Gene encoding putative LysM protein with GenBank accession number MT037016, and gene encoding LysM protein GenBank accession number: MT037017 was found only in *C*. *chloroleuca* and *C*. *solani*, respectively.*

**FIGURE 1:**
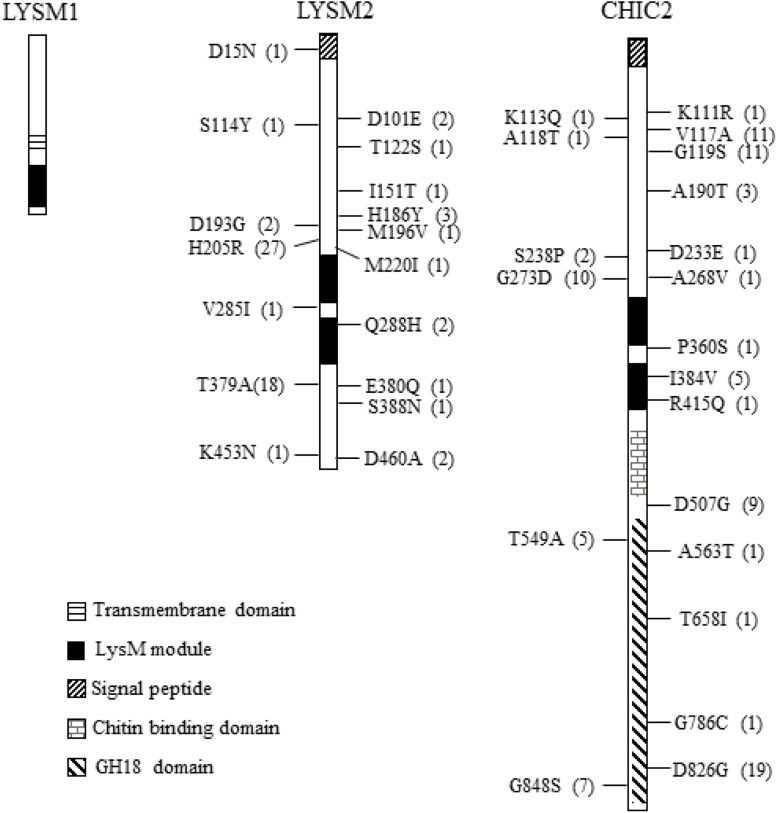
Non-synonymous nucleotide substitution in the *lysm1*, *lysm2*, and *chiC2* genes in *C*. *rosea* strains. LYSM1 has one LysM module between amino acid positions 127–171; LYSM2 and CHIC2 contain two LysM modules between amino acid positions 235–280, 289–339, and 309–354, 372–419, respectively. The single nucleotide polymorphism (SNPs) were compared among worldwide collection of 53 *C*. *rosea* strains. The number in parenthesis indicates the number of strains share the same nucleotide polymorphism. Detailed information of all SNPs reported in *lysm1*, *lysm2*, and *chiC2* can be found in Supplementary Table S2.

Analysis of translated amino acid sequences for the presence of conserved modules using SMART identified a single 45-amino-acid LysM module (PFAM: PFO1476; InterPro: IPR018392) between positions 127-171 and a 23-amino-acid transmembrane region between positions 87-109 for LYSM1. LYSM2 contained two LysM modules (46 and 50 amino acids, respectively) between positions 235-280 and 289-339, and is predicted to contain a signal peptide in the N-terminus (Figure 1). The CHIC2 protein, contained a 20-amino-acid N-terminal signal peptide, two LysM modules between positions 309–354 and 372–419, an additional chitin-binding (CBM18) module, and a catalytic glycoside hydrolase family 18 (GH18) module (Figure 1), conforming to the known structure of subgroup C-II killer toxin-like chitinases (Tzelepis et al., 2015). These results suggest that LYSM2 and CHIC2 are putatively secreted, and LYSM1 is a putative transmembrane protein.

The amino acid sequences of LYSM1, LYSM2 and CHIC2 were predicted to contain four, 22 and 32 Cys residues, respectively. From these, three Cys residues were always present in each LysM module and one Cys was always located close, but outside, to the LysM modules suggesting that these LysM modules belonged to the fungal-specific group. Based on our data, LYSM2 is predicted to be LysM effector, and LYSM1 and CHIC2 are LysM-containing proteins (de Jonge and Thomma, 2009). A phylogenetic analysis of the five individual LysM modules from *C. rosea*, together with LysM modules from other species, was not able to distinguish between internal tandem gene duplication or exon shuffling as the cause of the dual LysM modules present in LYSM2 and CHIC2, due to very low bootstrap support (≤62%, Supplementary Figure S1). In contrast, LysM modules number four, five, six and seven from *T. atroviride* TAL6 clustered together with 97% bootstrap support, indicating internal gene duplication.

Genome sequence analysis of four different *Clonostachys* spp. (Broberg et al., unpublished) revealed that *C*. *byssicola*, *C*. *rhizophaga*, *C*. *chloroleuca*, and *C*. *solani* each contained three genes orthologous to *C*. *rosea’s lysm1*, *lysm2*, and *chiC2*. GenBank accession number is provided in Supplementary Table S4. *C*. *chloroleuca* and *C*. *solani* also contained one additional gene each, predicted to encode a fungal/bacterial (GenBank accession number: MT037016) and fungal-specific (GenBank accession number: MT037017) LysM protein, respectively (Table 1). Sequence alignments of the predicted LysM proteins from five *Clonostachys* species showed that LYSM1 and CHIC2 were highly conserved between species (98 and 95% amino acid identity, respectively), while LYSM2 showed a lower level (75%) of amino acid identity.

### Analysis of LysM Gene Sequence Polymorphisms in *Clonostachys rosea*

We assessed DNA sequence variation of LysM genes in 53 genome-sequenced strains of *C*. *rosea* (Broberg et al., 2018). All these strains contain one copy of *lysm1*, *lysm2* and *chic2*. Nine synonymous SNPs but no non-synonymous SNPs were identified in *lysm1* in 17 strains (Figure 1 and Supplementary Table S2). In *lysm2*, 14 synonymous and 17 non-synonymous SNPs were identified across three exons involving 38 strains (Figure 1 and Supplementary Table S2). Only four SNPs (three synonymous, one non-synonymous) were located in the two LysM modules (Figure 1 and Supplementary Table S2). In *chiC2*, 57 synonymous and 20 non-synonymous SNPs were found across two exons of *chiC2* in 38 strains, five of which were present in the LysM modules. Two of these SNPs were non-synonymous, I384V and R415Q (Figure 1 and Supplementary Table S2). A MDS analysis revealed a correlation between allele distribution and geographic origin of strains for *lysm2* and *chic2*, but not for *lysm1* (Supplementary Figure S2). GARD analysis indicated (*P* < 0.001) one potential recombination event at position 192 in *lysm1* and another potential recombination event at position 531 in *chiC2* (Supplementary Figure S2). REL analysis indicated one site evolving under positive selection (Bayes factor = 46) at codon 205 in *lysm2* and one site (Bayes factor = 699) at codon 826 in the predicted GH18 module of *chiC2* (Figure 1 and Supplementary Figure S2).

### Gene Expression Analysis

Gene expression analyses of *lysm1* and *lysm2* were carried out in *C*. *rosea* strain IK726 under conditions relevant for *lysm* gene expression like fungal-fungal and fungal-plant interactions, in culture media amended with chitin or peptidoglycan as a sole carbon source and during conidia germination.

**FIGURE 2:**
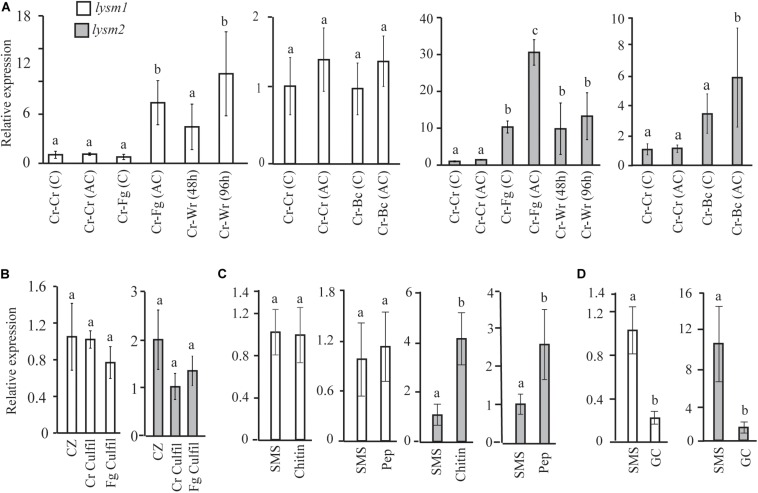
Expression analysis of *lysm1* and *lysm2* in *C*. *rosea*. **(A)** During *in vitro* interaction with *B*. *cinerea* (Cr-Bc) or *F*. *graminearum* (Cr-Fg) at two time points: at contact (C) and at 24 h after contact (AC) stage, and wheat root (Cr-Wr) at 48 and 96 hpi. *C*. *rosea* confronted with itself (Cr-Cr) was used as control. **(B)** Gene expression in self (Cr Culfil) or *F*. *graminearum* (Fg Culfil) culture filtrates, mycelia grown in liquid Czapek-dox (CZ) was used as control. **(C)** Gene expression analysis in *C*. *rosea* mycelia grown in liquid SMS medium amended with 1% colloidal chitin or 0.1% peptidoglycan (Pep) as a sole carbon source. SMS medium amended with 1% glucose was used as control. **(D)** Gene expression analysis during conidial germination. Liquid SMS with 1% glucose was used for the conidial germination. C. *rosea* mycelia grown in SMS with 1% glucose was used as control. Relative expression level based on RT-qPCR was calculated as the ratio between the target *lysm* gene and β-tubulin gene using 2^–ΔΔ*Ct*^ method (Livak and Schmittgen, 2001), and compared with the respective control. Error bar represent standard deviation based on three or five biological replicates. Statistically significant differences (*P* ≤ 0.05) in gene expression between treatments were determined using Fisher’s exact test or *T*-test and are indicated by different letters.

*lysm1* and *lysm2* gene expression was investigated in *C*. *rosea* during dual plate interaction with the fungal prey *B. cinerea* or *F. graminearum* at two time points: at the mycelial contact stage and at 24 h after mycelial contact. Gene expression data showed no significant difference in *lysm1* expression in *C*. *rosea* at neither contact nor after contact stages during interaction with *B*. *cinerea*, compared with a *C*. *rosea* self-interaction control (Cr-Cr); however, a significant 7.4-fold (*P* ≤ 0.012) increase in *lysm1* expression was recorded during interaction with *F*. *graminearum* at 24 h after mycelial contact, compared with Cr-Cr (Figure 2A). Expression of *lysm2* was 5.8- fold higher (*P* ≤ 0.023) during interaction with *B*. *cinerea* after the mycelial contact stage, and with *F*. *graminearum* at both contact (10.3-fold) and after contact (30.8-fold) stages, compared with Cr-Cr (Figure 2A).

For *lysm1* and *lysm2* gene expression analyses in *C*. *rosea* during interaction with wheat roots, samples were harvested at 48 and 96 hpi. Expression of *lysm1* was significantly higher (10.4-fold; *P* ≤ 0.012) only during colonization of wheat roots 96 hpi, while expression of *lysm2* was 9.8-and 13.2-fold higher (*P* ≤ 0.023) during colonization of wheat roots at both time points, compared with Cr-Cr (Figure 2A).

*lysm1* and *lysm2* expression analysis was performed in culture filtrates from *F*. *graminearum* and from *C*. *rosea* itself to investigate the effect of secreted factors including the chitinases on expression of LYSM genes. The culture filtrate from *C*. *rosea* itself was used to investigate the possible role of LYSM proteins in protection against self-produced chitinases, and also to investigate common or species specific response in *lysm1* and *lysm2* expression. No differences in *lysm1* or *lysm2* expression were found in *C*. *rosea* mycelia grown in self-culture filtrates or *F*. *graminearum* culture filtrates, compared with the control treatment (Figure 2B).

Expression of *lysm1* and *lysm2* was further assessed in *C*. *rosea* grown in liquid SMS medium amended with 1% colloidal chitin or 0.1% peptidoglycan (Sigma-Aldrich, St. Louis, MO, United States) and compared to SMS medium with 1% glucose. Expression of *lysm2*, but not *lysm1*, was significantly higher (*P* ≤ 0.009) when *C*. *rosea* was grown in SMS medium containing colloidal chitin (4.0-fold) or peptidoglycan (2.6-fold) as a sole carbon source, compared with SMS medium with glucose (Figure 2C).

Gene expression of *lysm1* and *lysm2* in germinated conidia in SMS with 1% glucose was compared with the *C*. *rosea* mycelia grown under same conditions. Expression of *lysm1* and *lysm2* was 4.5-fold and 7.1-fold low (*P* ≤ 0.019), respectively, during conidial germination compared with expression in *C*. *rosea* mycelia (Figure 2D). Expression of *lysm1* and *lysm2* was observed in all tested conditions.

### Generation of Gene Deletion and Complementation Strains

To characterize the biological role of LYSM 1 and LYSM2 in *C*. *rosea*, single *lysm1* and *lysm2*, and double Δ*lysm1*Δ*lysm2* deletion strains were generated through ATMT. Single *lysm1* and *lysm2* deletion mutants were generated by replacing *lysm1* and *lysm2* with the hygromycin resistance gene cassette hygB (Supplementary Figure S3A). A double Δ*lysm1*Δ*lysm2* deletion strain was generated by replacing *lysm2* with the sulfonylurea resistance gene selection cassette (ILV1*)* in a Δ*lysm1* strain. Successful gene replacement in hygromycin-/sulfonylurea-resistant transformants were confirmed by PCR using primers located within the hygB/ILV1 cassette, together with primers located upstream or downstream of the construct (Supplementary Figure S3A) as described in our previous works (Dubey et al., 2013a, b). The expected size of PCR fragments were amplified in Δ*lysm1*, Δ*lysm2* and Δ*lysm1*Δ*lysm2* strains, while no amplification was observed in the WT (Supplementary Figures S3B–D). Furthermore, RT-PCR experiments using primers specific to the *lysm1* and *lysm2* sequence demonstrated the complete loss of *lysm1*and *lysm2* transcript in each individual and double deletion mutant, while expression of *lysm1* and *lysm2* were detected in WT (Supplementary Figures S3B,C).

Single Δ*lysm1* and Δ*lysm2* strains were complemented with *lysm1* and *lysm2* through ATMT, respectively. Successful integration of the ILV1 selection cassette in mitotically stable mutants was confirmed by PCR amplification of ILV1. RT-PCR from randomly selected ILV1 positive Δ*lysm1* and Δ*lysm2* strains using *lysm1*- and *lysm2*-specific primer pairs, demonstrated restored *lysm1* or *lysm2* transcription in *lysm1* and *lysm2* complemented (Δ*lysm1*+; Δ*lysm2*+) strains, respectively, while no transcripts were detected in the parental deletion strains (Supplementary Figures S3E,F).

### Phenotypic Analyses

#### Deletion of *lysm1* or *lysm2* Affects Conidial Germination and Mycelial Integrity

**FIGURE 3:**
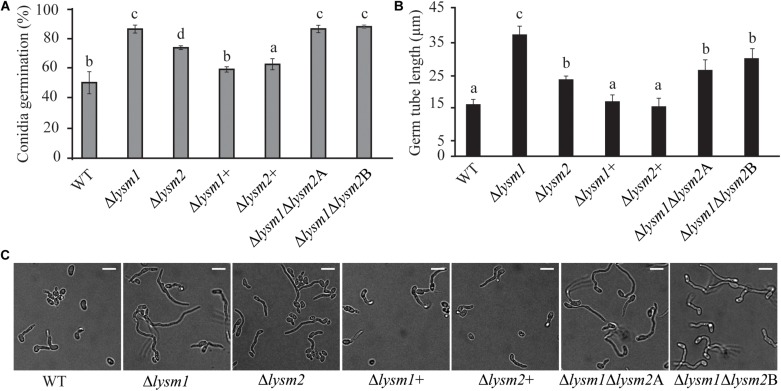
Analysis of conidial germination and germ tube length 8 h post inoculation on half-strength PDA medium. **(A,C)** Frequency of germinating conidia was determined by counting the number of germinating and non-germinating conidia. **(B,C)** Germ tube length was measured with the ImageJ image analysis software (Schneider et al., 2012). Scale bar: 10 μm. Error bars represent standard deviation based on four biological replicates. Different letters indicate statistically significant differences (*P* ≤ 0.05) within the experiments based on Fisher’s exact test.

Deletion of *lysm1*, *lysm2*, or *lysm1lysm2* did not affect the mycelial growth rate and biomass production on PDA or in PDB medium 5 dpi, respectively. Conidial germination and germ tube length were determined after eight h and 24 h of incubation on PDA. After eight h of incubation, *lysm* deletion strains had significantly (*P* ≤ 0.007) higher conidial germination (1.5-fold – 1.7-fold) and germ tube length (1.5-fold – 2.3-fold) compared with the WT (Figures 3A–C). However, these differences were no longer detected (*P* ≥ 0.24) after prolonged incubation for 24 h (Supplementary Figure S4). The conidial germination of the Δ*lysm1* strain was significantly (*P* < 0.001) higher compared with the Δ*lysm2* strain (Figure 3A), while conidial germ tube length of the Δ*lysm1* strain was significantly (*P* < 0.001) higher compared with both the Δ*lysm2* and the Δ*lysm1*Δ*lysm2* strains (Figures 3B,C). Complementation strains Δ*lysm1*+ and Δ*lysm2*+ showed partial restoration of normal conidial germination levels, and complete restoration of germ tube length (Figures 3A–C).

**FIGURE 4:**
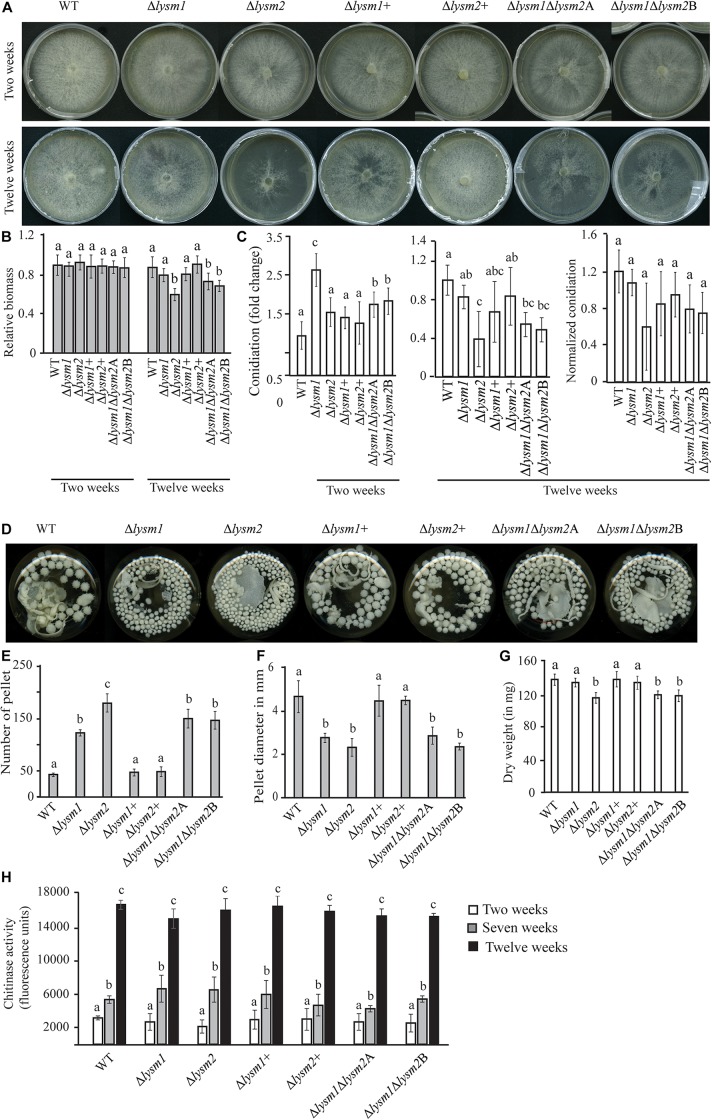
Phenotypic characterizations of *C*. *rosea* WT, deletion and complementation strains. **(A,B)** Deletion of *lysm2* affects hyphal protection. After 2 weeks of inoculation no visible difference in mycelial disintegration was found between the WT and the deletion strains. However, after 12 weeks of inoculation, mycelial disintegration was distinct in the Δ*lysm2* and Δ*lysm1*Δ*lysm2* deletion strains. **(C)** Conidiation of WT, *lysm* deletion and complementation strains on PDA medium after 2 and 12 weeks of inoculation. The number of conidia from each treatment was normalized to their respective mycelial biomass. **(D–F)** Deletion of *lys*m1 or *lysm2* affects the mycelial pellet formation in liquid media. **(G)** Biomass production in *C*. *rosea* WT and deletion strains 12 weeks post inoculation. Biomass production was determined measuring mycelial dry weight. **(H)** Chitinase activity analysis in culture filtrates from *C*. *rosea* WT and the deletion strains 2 weeks, 7 weeks and 12 wpi in liquid SMS medium with 1% glucose. Error bars represent standard deviation based on five biological replicates. Different letters indicate statistically significant differences based on Tukey HSD method at the 95% significance level.

Mycelial colony morphology on PDA was recorded weekly during a 13-week period. An accelerated mycelial disintegration/lysis phenotype leading to significant (*P* ≤ 0.034) reduction in mycelial biomass was observed 6 weeks post inoculation (wpi) and onward in the Δ*lysm2* and Δ*lysm1*Δ*lysm2* deletion strains, compared with the WT strain (Supplementary Figure S5). Twelve wpi, the Δ*lysm2* and Δ*lysm1*Δ*lysm2* strains displayed severe (*P* ≤ 0.005) disintegration/lysis of the colony and reduction in the biomass compared to that of the WT (Figures 4A,B and Supplementary Figure S5). The experiment was repeated three times with the same result.

Conidial production of *C*. *rosea* WT and *lysm* deletion strains was determined two and 12 wpi by counting the conidia harvested in equal amounts of water. Two wpi, 1.6-fold – 2.6-fold higher (*P* ≤ 0.001) conidial numbers were recorded in all *lysm* deletion strains compared with the WT (Figure 4C), indicating that deletion of *lysm* accelerated conidial production and hence increased conidial yield in the *lysm* deletion strains 2 wpi. However, the differences in conidial yield between the WT and the Δ*lysm1* strain were diminished 12 wpi, and were significantly (*P* ≤ 0.001) lower in the Δ*lysm2* and the Δ*lysm1*Δ*lysm2* strains compared with the WT (Figure 4C). In order to confirm whether reduced number of conidia in the Δ*lysm2* and Δ*lysm1*Δ*lysm2* strains was related with their mycelial disintegration, the number of conidia produced in the WT and the deletion strains was normalized with their respective mycelial biomass at 12 wpi. After normalization, no significant (*P* ≥ 0.368) difference in number of conidia was found between the WT and the deletion strains (Figure 4C).

A similar experiment was performed using submerged liquid cultures, where conidia from the WT and *lysm* mutant strains were inoculated in SMS medium with 1% glucose as a carbon source and incubated at a rotary shaker (150–200 rpm) for 12 weeks. Already after one wpi, a clear difference in size of the mycelial pellets was observed between the WT and the deletion strains. The mycelial pellets of Δ*lysm1*, Δ*lysm2*, and Δ*lysm1*Δ*lysm2* were 39% – 50% smaller in size, but more numerous, compared with the WT and the complemented strains (Figures 4D–F). In addition, a visible hyphal disintegration was observed in the cultures of the WT and the deletion strains over time, which became more pronounced in cultures of the Δ*lysm2* and the Δ*lysm1*Δ*lysm2* strains compared with the WT. In addition, mycelial biomass of the Δ*lysm2* and the Δ*lysm1*Δ*lysm2* strains was 16–20% reduced (*P* ≤ 0.001) compared with the WT 12 wpi (Figure 4G).

In order to test whether biomass reduction were associated with the hydrolytic activity of secreted enzymes, chitinase activity was measured in the culture filtrates from the WT and the deletion strains 2, 7, and 12 wpi. Chitinase activity increased significantly (*P* ≤ 0.020) over time for all strains (Figure 4H). Chitinase activity was also compared between the WT and the deletion strains. No significant differences in chitinase activity were recorded in the culture filtrates from the WT and the deletion strains two, seven and 12 wpi (Figure 4H).

#### LYAM1 and LYSM2 Contribute to Hyphal Protection Against Chitinases

**FIGURE 5:**
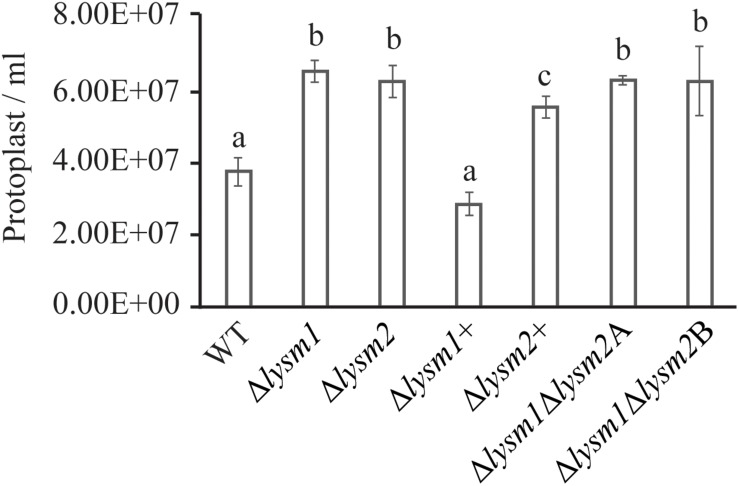
Cell wall protection assay of *C*. *rosea* strains against snailase enzyme mix. Protoplast release from *C*. *rosea* and deletion strains was counted after 60 min of incubation. The experiment was performed in five replicates. Different letters indicate statistically significant differences based on Fisher’s exact test at the 95% significance level.

Protoplast release has been used to measure the cell wall stability and hyphal protection against the hydrolytic activity of chitinases (Cen et al., 2017; Romero-Contreras et al., 2019). We evaluated the role of LYSM1 and LYSM2 in *C*. *rosea* hyphal protection by measuring the protoplast release after treating the mycelia with lytic enzyme mix including the chitinases. The result showed higher number (*P* ≤ 0.002) of protoplast were released from the mycelia of Δ*lysm1*, Δ*lysm2* and Δ*lysm1*Δ*lysm2* strains compared to the WT strains (Figure 5). No significant difference was found in protoplast release between either of single LYSM deletion strains and double deletion strains. Complementation strains Δ*lysm1*+ and Δ*lysm2*+ showed complete and partial restoration of protoplast release, respectively. The result suggest that LYSM1 and LYSM2 contribute to hyphal protection against exogenous chitinases.

#### Deletion of *lysm2* Reduces Antagonistic Ability Toward *Botrytis cinerea*

**FIGURE 6:**
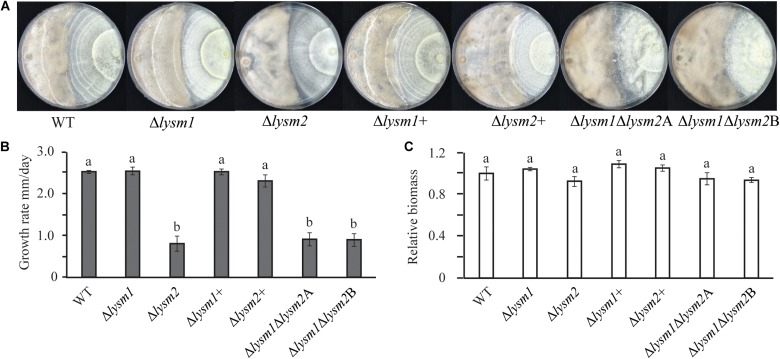
Mycoparasitism analyses of *C*. *rosea* (right side in the plate) strains against *B. cinerea* (left side in the plate). **(A)** The Δ*lysm2* and Δ*lysm1*Δ*lysm2* strains showed reduced ability to grow on *B*. *cinerea*. **(B)** Growth rate (overgrow) of *C*. *rosea* WT and deletion strains on *B*. *cinerea*. The growth of *C*. *rosea* on *B*. *cinerea* was measured from the point of mycelial contact. **(C)** Mycelial biomass of *C*. *rosea* WT and deletion strains 3 weeks post inoculation. Mycelial biomass was quantified by measuring the pixel intensity of the photographs using ImageJ image analysis software (Schneider et al., 2012). The experiment was performed in four biological replicates and photographs of representative plates were taken 3 weeks post inoculation.

Antagonism of *C*. *rosea* WT and deletion strains was measured using a dual-culture-plate confrontation assay and a culture-filtrate assay. During dual-culture interactions, *C*. *rosea* initially reduces the mycelial growth of the fungal prey, and then, after mycelial contact, starts to overgrow and conidiate on the mycelia of the prey fungus (i.e., *F*. *graminearum* and *B*. *cinerea* (Dubey et al., 2014a, b, 2016). No differences in growth rate or mycelial biomass of *F*. *graminearum* or *B*. *cinerea* were recorded during *in vitro* dual-plate confrontation with either single or double *lysm* deletion strains, compared with the WT (Supplementary Figure S6). Similarly, no differences in growth rate or mycelial biomass between *C*. *rosea* WT and *lysm* deletion strains were measured during the same conditions (Supplementary Figure S6). However, on average, a 3.0-fold reduced (*P* ≤ 0.001) growth rate on *B*. *cinerea* mycelia (overgrowth rate) was observed in the Δ*lysm2* and the Δ*lysm1*Δ*lysm2* strains compared to the growth rate of the WT (Figures 6A,B). A similar experiment against *F*. *graminearum* showed no difference in antagonism between the WT and the single or double *lysm* deletion strains (Supplementary Figure S6). In order to study whether the reduced ability to overgrow *B*. *cinerea* was related with a reduction in mycelial biomass of the Δ*lysm2* and the Δ*lysm1*Δ*lysm2* strains, mycelial biomass of *C*. *rosea* WT and *lysm* deletion strains was quantified. No significant differences in mycelial biomass was recorded between the WT and *lysm* deletion strains (Figure 6C).

In order to investigate a role of LYSM protein in tolerance against secreted chitinases, mycelial biomass of *C*. *rosea* WT and *lysm* deletion strains was measured in the culture filtrates from the prey fungus *B. cinerea* or *F. graminearum*. Similar to the result from the dual-culture experiment, no significant differences in mycelial biomass was found between the WT and the *lysm* deletion strains when grown in culture filtrates from either *B. cinerea* or *F. graminearum* (Supplementary Figure S6).

### LYSM1 and LYSM2 Contributes to Biocontrol of *Botrytis cinerea* and *Fusarium graminearum*

**FIGURE 7:**
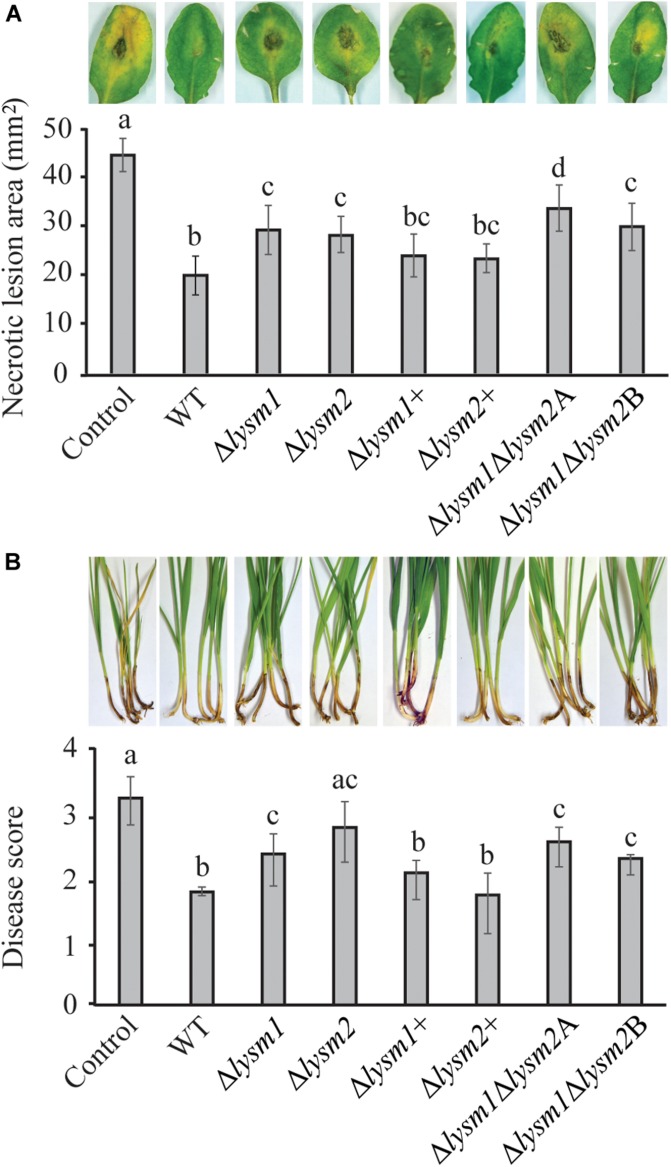
*In vivo* bioassay to test the biocontrol ability of *C*. *rosea* strains against *B. cinerea* and *F. graminearum*. **(A)** Measurement of *B*. *cinerea* necrotic lesions on detached leaves of *A*. *thaliana* plants. The leaves were inoculated with *C*. *rosea* strains 60 min before application of *B*. *cinerea* and allowed to interact for 60 h. Only pathogen inoculated leaves were used as control. Necrotic lesion area was measured under the microscope using DeltaPix camera and software. The exprement was performed in five biological replicates with six leaves in each replicate in each treatment. **(B)** Foot rot disease on wheat using sand seedling test. Seedlings were harvested 3 weeks post inoculation and disease symptoms were scored on 0–4 scale. The experiment was performed in five biological replicates with 15 plants in each replicate. Different letters indicate statistically significant differences (*P* ≤ 0.05) within experiments based on the Fisher’s exact test.

The biocontrol performance of *C*. *rosea* WT and *lysm* deletion strains against the necrotrophic pathogen *B*. *cinerea* was determined on detached *Arabidopsis thaliana* leaves. Significant (*P* ≤ 0.022) increases of 28.6% – 40% in necrotic lesion area were measured on leaves pre-inoculated with conidia from the Δ*lysm1*, Δ*lysm2* and the Δ*lysm1*Δ*lysm2* strains, compared to the necrotic lesion area on leaves pre-inoculated with *C*. *rosea* WT spores (Figure 7A). However, complementation strains Δ*lysm1*+ and Δ*lysm2*+ showed partial restoration of the WT phenotype.

The biocontrol ability of *C. rosea* strains was further assessed against *F*. *graminearum* foot rot disease on wheat plants using a growth chamber sand seedling test. Our result showed 21% – 43% increase (*P* ≤ 0.031) of disease severity in wheat seedlings previously seed coated with conidia from the Δ*lysm1*, Δ*lysm2* and the Δ*lysm1*Δ*lysm2* strains, compared with seedlings from seeds coated with WT *C. rosea* (Figure 7B). In all cases, complementation of *lysm* deletions strains restored the biocontrol phenotypes to WT levels. With the exception for biocontrol of fusarium foot rot by the Δ*lysm2* strain, all other *lysm* deletion strains did not completely abolish the biocontrol effect as disease symptoms were significantly (*P* ≤ 0.006) milder compared with the symptoms from control treatments (pathogen inoculation without any *C. rosea* strain) (Figures 7A,B).

With the exception for biocontrol of *B. cinerea* by the Δ*lysm1*Δ*lysm2*A strain, no significant differences in biocontrol ability were recorded between any of the *lysm* deletion strains against the tested pathogens.

### LYSM1 and LYSM2 Are Jointly Required for Suppression of Defense Gene Expression in Wheat

**FIGURE 8:**
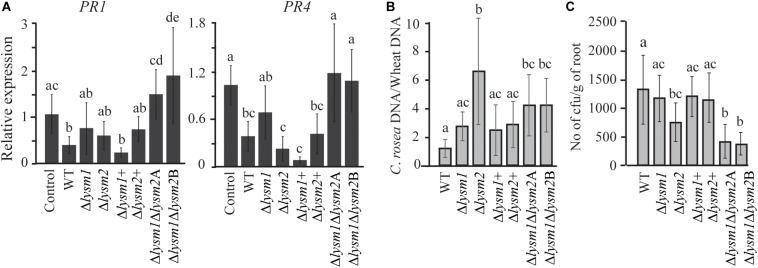
Expression analysis of defense related genes and root colonization assay in wheat. **(A)** Gene expression analysis of defense response related genes in wheat roots colonized by *C*. *rosea* strains. Total RNA isolated from wheat roots 5 days post inoculation of WT and Δ*lysm* strains and was used for RT-qPCR. Expression level of *PR1* and *PR4* genes was normalized to expression of the wheat β-tubulin (Marshall et al., 2011). Relative expression level based on RT-qPCR was calculated using 2^–ΔΔ*Ct*^ method (Livak and Schmittgen, 2001), and compared with the water inoculated control. **(B)** Determination of *C*. *rosea* root colonization in wheat roots, 5 days post inoculation, by quantifying DNA level using RT-qPCR. *C*. *rosea* colonization is expressed as the ratio between *C*. *rosea* DNA and wheat DNA. For DNA quantification *actin* and *Hor1* were used as target gene for *C*. *rosea* and wheat, respectively. **(C)** Internal root colonization by *C*. *rosea* strains. Surface sterilized roots were homogenized in phosphate buffer and serial dilutions were plated on Rose Bengal selection plates under sterile condition at 25°C. Colony forming units (cfus) were counted 3 days post plating. Statistically significant differences (*P* ≤ 0.05) in gene expression between treatments were determined using Fisher’s exact test and are indicated by different letters. Error bars represent standard deviation based on five biological replicates.

To investigate whether LYSM1 and LYSM2 interfere with plant defense responses, expression of wheat defense response genes *PR1* and *PR4* (Marshall et al., 2011) was measured with RT-qPCR in wheat roots inoculated with *C*. *rosea* WT, *lysm* deletion and complementation strains. Our data showed a significant 2.63-fold and 2.73-fold (*P* ≤ 0.032) suppression in expression of *PR1* and *PR4* genes, respectively, in wheat roots colonized by *C*. *rosea* WT, compared with the non-inoculated control (Figure 7A). However, the double deletion strains Δ*lysm1*Δ*lysm2* failed to suppress expression of both genes in wheat roots to *C. rosea* WT levels, in contrast with the single Δ*lysm1* and Δ*lysm2* mutants (Figure 8A).

Furthermore, in order to analyze the contribution of LYSM1 and LYSM2 in root colonization, the biomass of *C*. *rosea* strains on wheat roots was measured 5 dpi by quantifying the ratio between *C*. *rosea* DNA and wheat DNA with qPCR. Significantly higher *C*. *rosea*/wheat DNA ratios were detected on wheat roots inoculated with the Δ*lysm2* strain (5.5-fold; *P* < 0.001) or the Δ*lysm1*Δ*lysm2* strains (4.2-fold; *P* ≤ 0.026), compared with roots inoculated with the WT (Figure 7B), suggesting increased biomass of the Δ*lysm2* and Δ*lysm1*Δ*lysm2* strains, compared with the WT. No significant differences in *C*. *rosea*/wheat DNA ratio were found between WT and Δ*lysm1*, Δ*lysm1*+ or Δ*lysm2*+ inoculated wheat roots (Figure 8B).

Colony forming units (cfus) of surface sterilized roots were determined to measure the internal root colonization levels of *C*. *rosea* WT and deletion strains. A significant reduction (*P* < 0.001) of 47 or 70% in cfus from the roots inoculated with the Δ*lysm2* or the Δ*lysm1*Δ*lysm2* strains were found, compared to the roots inoculated with the WT (Figure 8C). No significant differences in cfus were found between the WT and the Δ*lysm1*, Δ*lysm1*+ or Δ*lysm2*+ inoculated wheat roots (Figure 8C).

### Expression Analysis of *lysm1*, *lysm2*, and *chiC2* in the WT and the *lysm* Deletion Strains

In order to study if deletion of *lysm1* results in regulatory feed-back loops that influence expression levels of *lysm2* and vice versa, gene expression analysis was performed using RNA extracted from mycelia of WT, Δ*lysm1* and Δ*lysm2* strains. RT-qPCR gene expression analysis revealed no significant (*P* = 0.26) difference in *lysm1* expression between mycelia of the WT and the Δ*lysm2* strain (Supplementary Figure S7A), and similarly, no significant (*P* = 0.053) difference in *lysm2* expression was observed between the WT and the Δ*lysm1* strain (Supplementary Figure S7B). Expression levels of *chiC2* were also analyzed in *C*. *rosea* WT, and deletion strains including the complementation and the double deletion strains. Similar to *lysm1* and *lysm2*, no significant difference (*P* ≥ 0.245) in expression of *chiC2* was found between the WT and the deletion strains (Supplementary Figure S7C).

## Discussion

Filamentous fungi generally contain multiple genes coding for LysM proteins (de Jonge and Thomma, 2009; Cen et al., 2017), especially in plant pathogens such as *F*. *graminearum* (13 genes), *F*. *oxysporum* (16 genes), *F*. *verticillioides* (17 genes) and in insect pathogens such as *B. bassiana* (12 genes) and *Metarhizium robertsii* (13 genes) (de Jonge and Thomma, 2009; Cen et al., 2017). Even certain mycoparasitic and plant beneficial fungi, such as *T*. *atroviride* and *T*. *virens*, contain high numbers of LysM-encoding genes (12 and 18 genes, respectively). Furthermore, the diversity in LysM gene copy number can vary considerably even between closely related fungal species (de Jonge and Thomma, 2009; Akcapinar et al., 2015; Cen et al., 2017; Kombrink et al., 2017). For instance, genomes of plant root colonizing and mycoparasitic *Trichoderma* spp. possess an increased number of genes encoding secreted LysM proteins compared with primarily saprophytic *Trichoderma* spp. (Kombrink and Thomma, 2013; Mendoza-Mendoza et al., 2018). Similarly, the generalist insect pathogen *M*. *robertsii* contains 13 LysM genes, whereas only four genes are present in the specialist species *M*. *acridum* (Cen et al., 2017). In addition to gene copy number variation, previous studies also report high sequence variation (sometimes driven by positive selection) and modular structure in LysM proteins (Akcapinar et al., 2015; Cen et al., 2017; Kombrink et al., 2017). Taken together, this indicates that ecological niche adaptation drives the evolution of the fungal LysM gene family through a birth-and-death process followed by sequence diversification (Wapinski et al., 2007; Marshall et al., 2011; Cen et al., 2017).

The relatively low number of three LysM genes in the mycoparasitic and plant beneficial fungus, *C. rosea*, therefore suggests important ecological differences between *C. rosea* and related hypocrealean plant and insect pathogens. Especially, the difference between *C. rosea* and *Trichoderma* spp. is striking, given the apparent similarity in their ecology as necrotrophic mycoparasites and plant beneficial symbionts, and suggests that the role of LysM proteins in *C. rosea* may differ to those in *Trichoderma* spp. One important aspect of the LysM gene family in *C*. *rosea* is the predicted membrane localization of LYSM1, which is rare in fungi and may suggest a function as a receptor.

Analysis of sequence polymorphism showed that *lysm1* is highly conserved, both between *C*. *rosea* strains and between *Clonostachys* species, indicating a conserved function. In contrast, the higher levels of sequence polymorphisms and the presence of codons predicted to evolve under positive selection suggests different evolutionary trajectories of *lysm2* and *chiC2*, compared with *lysm1*. However, these results should be interpreted with care as the presence of recombination may interfere with the analysis of molecular evolution. Our results are in line with previous data from the leaf pathogen *Z*. *tritici* where codons in *MgLysM1* and *MgLysM3* were shown to evolve under positive selection (Marshall et al., 2011). Different degrees of sequence polymorphisms were also reported in core LysM effectors in the soil borne pathogen, *V*. *dahlia*, while no evidence of positive or negative selection was found (Kombrink et al., 2017).

What can then be the function of LYSM1 and LYSM2? The expansion of the LysM gene family in plant, insect pathogenic and mycoparasitic fungi is hypothesized to be associated with their important role in dampening the host defense response by chitin sequestration, consequently avoiding recognition during the interactions with their hosts (de Jonge and Thomma, 2009; Kombrink and Thomma, 2013; Romero-Contreras et al., 2019). This function has been proven for several LysM effectors, for instance Ecp6 from the tomato leaf mold pathogen, *Cladosporium fulvum* (de Jonge et al., 2010), Mg3LysM from the septoria leaf blotch fungus, *Z*. *tritici* (Marshall et al., 2011), Slp1 from the rice blast pathogen, *Magnaporthe oryzae* (Mentlak et al., 2012), ChELP1 and ChELP2 from the Brassicaceae anthracnose fungus, *Colletotrichum higginsianum* (Takahara et al., 2016), Vd2LysM from the soil borne vascular wilt fungal pathogen, *V. dahliae* (Kombrink et al., 2017), Blys2 and Blys5 in the entomopathogen, *B. bassiana* (Cen et al., 2017). Other LysM effectors are reported to protect the fungal cell wall against chitinases produced by the host, for example in the case of Mg1LysM and Mg3LysM from *Z. tritici* (Marshall et al., 2011), and Blys5 from *B. bassiana* (Cen et al., 2017). Furthermore, LysM effector Tal6 from the mycoparasitic fungus *T. atroviride* has been implicated in mycoparasitic interaction and modulation of host defense response (Romero-Contreras et al., 2019).

The repressed expression of *C. rosea lysm1* and *lysm2* in germinating conidia, compared with filamentous growth, fits well with a possible function as an inhibitor of spore germination. This hypothesis is further supported by the fact that *lysm1* deletion strains displayed increased conidiation, conidial germination and germ tube length, not only compared with the WT but also compared with the Δ*lysm2* strain. This result is in line of previous findings from *T. atroviride*, where external application of a purified LysM protein TAL6 displayed a strong inhibitory effect on spore germination in *T. atroviride* and *T. reesei*, but not in more distantly related fungi, providing evidence for a role of LysM proteins in regulating developmental processes in fungi (Seidl-Seiboth et al., 2013). Based on the lack of binding of TAL6 to unmodified chito-oligosaccharides or fungal cell wall material, Seidl-Seiboth and co-authors hypothesized that the germination inhibitor function of TAL6 may be exerted through binding of modified chito-oligosaccharides that acts as autoregulatory signaling molecules (Seidl-Seiboth et al., 2013). In the sporulating bacterium, *Bacillus subtillis*, the two LysM proteins safA and SpoVID localize to the cortex-coat interface of developing spores and interacts with each other and with coat components to induce formation of a protein complex that regulates spore formation (Costa et al., 2006). Although we, after several attempts, were not able to overexpress *C. rosea* LYSM1 and LYSM2 and test its chitin-binding capacity, we cannot exclude the possibility that LYSM proteins exerts its activity through direct binding of *C. rosea* cell wall chitin (Romero-Contreras et al., 2019). Irrespectively of the underlying mechanism, the observed alterations in mycelial pellet size in Δ*lysm1* strain suggest a disturbance in cell wall structure as it is known that fungal pellet formation is the result of spore aggregation that in turn depends on spore cell wall components and their interactions (Zhang and Zhang, 2016). Our results confirm the previous consideration that LysM proteins may act in physiological processes by affecting cell wall modification (Kombrink and Thomma, 2013).

The higher expression of *lysm2* during growth on chitin-containing media hints on a possible function of LYSM2 in protecting *C. rosea* hyphae against endogenous chitinases secreted during external chitin degradation. This hypothesis gain further support by the fact that *lysm2* is located adjacent to the killer toxin-like chitinase gene *chiC1* in the *C. rosea* genome (Karlsson et al., 2015; Broberg et al., 2018), and that the transcriptional analyses of *lysm2* in this study and *chiC1* in a previous study (Tzelepis et al., 2015) showed that both genes are co-induced in medium containing chitin as the sole carbon source. There are several examples of chitinase genes located physically close to co-regulated LysM protein-encoding genes (Gruber et al., 2011; Martinez et al., 2012), indicating an inter-related function of chitinases and LysM proteins (Gruber and Seidl-Seiboth, 2012).

The most distinctive phenotype in the *lysm2* gene deletions strain is the gradual increase in hyphal disintegration over time. Mycelial fragmentation and lysis due to secreted chitinases and other fungal cell wall-degrading enzymes has been demonstrated in other filamentous fungi like *Aspergillus nidulans*, *A. fumigatus* and *T. atroviride* (Carsolio et al., 1999; Yamazaki et al., 2007; Shin et al., 2009). However, the disintegration/lysis phenotype in the Δ*lysm2* strain is not related with increased production of chitinolytic enzymes, as the chitinase activity in Δ*lysm2* liquid cultures is similar to that of the WT. Similarly, this phenotype is not related to the susceptibility of the deletion strains to chitinases, as Δ*lysm1* strain that showed similar reduced hyphal protection against chitinases, only a weak disintegration phenotype can be observed. Instead, the most plausible explanation is that LYSM2 acts by protecting the *C. rosea* cell wall against its own chitinases.

Due to the role of LysM effectors in biotic interactions in plant pathogenic, entomopathogenic and mycoparasitic fungi, we hypothesized that LYSM1 and LYSM2 are involved in mycoparasitic interactions and plant root colonization by *C*. *rosea*. The significantly higher expression of *lysm1* and *lysm2* during interaction with *F*. *graminearum*, *B*. *cinerea*, and during the colonization of wheat roots, supports a role of the corresponding proteins in biotic interactions in *C. rosea*. We note that *lysm1* is expressed higher later during the interactions compared with *lysm2*, perhaps reflecting the putative plasma membrane-localization of LYSM1 that requires close interaction for contact with external inducers like chitin derivatives.

Our *in vivo* bioassay experiment with Δ*lysm1* and Δ*lysm2* strains showed that LYSM1 and LYSM2 are both required for biocontrol of gray mold on *A. thaliana* caused by *B*. *cinerea* and of fusarium foot rot disease on wheat caused by *F*. *graminearum*. For LYSM1, the proposed function as a regulator of fungal development and conidial germination does not contradict the reduced biocontrol phenotype. In both assays, *C. rosea* was applied as conidia and any factor that interferes with the normal spore germination program may also influence the highly complex process of biological control. For LYSM2, it is not surprising if the proposed function in protecting the *C*. *rosea* cell wall against hydrolytic enzymes also influence biocontrol. Mycoparasitism and biocontrol is a highly complex process that requires signaling between the two combatants, secretion of extracellular enzymes and secondary metabolites, and protection against the counterattack from the fungal prey (Karlsson et al., 2018). Therefore, it is not difficult to envision that a compromised cell wall in the Δ*lysm1* and Δ*lysm2* strains, exemplified by the disintegration and pellet size alteration phenotypes, will have a negative effect on biocontrol in *C. rosea*. From a more mechanistic aspect, secreted hydrolytic enzymes including chitinases are also produced by the prey fungus during competitive fungal-fungal interactions (Langner and Göhre, 2016). However, the lack of regulation of *lysm1* and *lysm2* gene expression in culture filtrates from *F*. *graminearum*, together with the lack of reduced growth of *lysm* deletion strains in culture filtrates from *B*. *cinerea* and *F*. *graminearum*, suggests that the reduced biocontrol ability is not primarily associated with secreted factors from the fungal prey. For LYSM2, the failure to overgrow *B. cinerea* in dual plate assays, but not *F. graminearum*, shows a certain level of specificity in LYSM2 function. This may be due to protection against fungal cell wall-degrading enzymes from *B. cinerea* or *C. rosea* itself that are specifically induced during their interaction, but not during growth in single cultures. Similar results showing host-specificity during mycoparasitism were reported previously in a study involving another mycoparasitic fungus, *T. virens*, in which deletion of the MAP kinase genes *TmkA* or *TmkB* resulted in a mutant with reduced ability to overgrow *Sclerotium rolfsii*, but not *Rhizoctonia solani* and *Pythium ultimum* (Kumar et al., 2010). Furthermore, *nox1*-overexpressed mutants of *T. harzianum* showed higher mycoparasitic activity toward *P. ultimum*, but not against *B. cinerea* (Montero-Barrientos et al., 2011).

Based on the expression analysis of the defense response marker genes *PR1* and *PR4* in wheat roots, we show that the *C*. *rosea* WT strain is able to suppress the pattern-triggered immunity (PTI) response in wheat. This is in line with previous findings where a transient suppression in peroxidase (higher expression during plant defense) activity was reported in *C*. *rosea*-treated wheat coleoptiles and roots (Roberti et al., 2008). Similarly, biocontrol *Trichoderma* spp. transiently suppresses PTI in *A*. *thaliana* by down-regulating genes associated with salicylic acid and jasmonic acid-related genes, and genes coding for plant cytochrome P450 monooxygenases that facilitate synthesis and metabolism of antimicrobial compounds (Morán-Diez et al., 2012; Brotman et al., 2013). Suppression of the host PTI response to establish root colonization has been shown in several plant beneficial microbes (Zamioudis and Pieterse, 2012). Deletion of *lysm1* or *lysm2* does not influence this ability; however, the failure of the Δ*lysm1*Δ*lysm2* double deletion strains to suppress expression of *PR1* and *PR4* to the same extent as the WT strain provides an indication that both LYSM proteins jointly influence this ability. The increased ratio between *C. rosea* and wheat DNA during root surface colonization and reduced ability of internal root colonization of the Δ*lysm2* strain, shows that LYSM2 also influences the interaction of *C. rosea* with plants; although, the exact mechanism will require extensive microscopic investigations of the root colonization process (Saraiva et al., 2015). The role of the LYSM2 protein in internal root colonization is in line with several examples from plant pathogenic fungi where deletion of LysM effector genes reduces their ability to infect the host plant (de Jonge and Thomma, 2009; de Jonge et al., 2010; Marshall et al., 2011; Mentlak et al., 2012; Kombrink and Thomma, 2013; Takahara et al., 2016). The similarity between LYSM2 and previously reported LysM effectors from plant pathogenic fungi in terms of interaction with plants, is intriguing considering the fact that LysM effectors from plant pathogenic fungi belong to the fungal/bacterial group, while *C*. *rosea* LYSM2 has a different modular structure than classical LysM effectors and belong to the fungal-specific LysM group.

In summary, we here characterize two LysM proteins in the mycoparasitic fungus *C. rosea* and show that both proteins influence conidial germination and the stability of the hyphae. Both proteins also influence the ability of *C. rosea* to control plant pathogenic fungi. Although the phenotypes of LYSM1 and LYSM2 are partially overlapping, differences in gene expression, predicted modular structure and the severity of the phenotypes suggest different functions; LYSM1 primarily as a regulator of fungal development and LYSM2 primarily in protecting the cell wall against hydrolytic enzymes and interactions with plant.

## Data Availability Statement

All datasets generated for this study are included in the article/Supplementary Material.

## Author Contributions

MD, DJ, and MK conceived and designed the experiments. MD and HV performed the experiments. MD, MB, and MK analyzed the data. DJ and MK contributed to the reagents, materials, and analysis tools. MD and MK wrote the manuscript. All authors read and approved the manuscript.

## Conflict of Interest

The authors declare that the research was conducted in the absence of any commercial or financial relationships that could be construed as a potential conflict of interest.
