# Supplementary material for: LysM Proteins Regulate Fungal Development and Contribute to Hyphal Protection and Biocontrol Traits in Clonostachys rosea
Source: Front Microbiol. 2020 Apr 16;11:679. doi: 10.3389/fmicb.2020.00679 (PMC7176902; doi:10.3389/fmicb.2020.00679)
Supplement: Supplementary file 1 [file Table_1.DOCX]

| Fungal species | Protein ID | Number of LysM modules | Other module/ domain | Comments |
| --- | --- | --- | --- | --- |
| *Trichoderma atroviride* | GenBank ID: XP_013947368.1 | 7 | no | Tal6 |
|  | JGI Protein ID: 162581 | 4 | no |  |
|  | JGI Protein ID: 43321 | 3 | no |  |
|  | JGI Protein ID: 85797 | 3 | no |  |
|  | JGI Protein ID: 205877 | 2 | no |  |
|  | JGI Protein ID: 307020 | 2 | ChtBD1 (4) |  |
|  | JGI Protein ID: 31285 | 2 | no |  |
| *Trichoderma reesei* | JGI protein ID: 105336 | 4 | no |  |
|  | JGI protein ID: 54723 | 2 | no |  |
| *Trichoderma virens* | JGI protein ID: 124493 | 4 | no |  |
|  | JGI protein ID: 128781 | 4 | no |  |
|  | JGI protein ID: 201746 | 4 | no |  |
|  | JGI protein ID: 149422 | 4 | no |  |
|  | JGI protein ID: 128337 | 3 | no |  |
|  | JGI protein ID: 66683 | 2 | no |  |
|  | JGI protein ID: 200487 | 2 | no |  |
| *Fusarium graminearum* | FGSG_12918.3 | 5 | no |  |
|  | FGSG_09119.3 | 3 | no |  |
|  | FGSG_10563.3 | 2 | no |  |
|  | FGSG_02255.3 | 1 | no |  |
| *Fusarium solani* | JGI protein ID: 56796 | 4 | no |  |
|  | JGI protein ID: 42325 | 4 | no |  |
|  | JGI protein ID: 90567 | 1 | no |  |
|  | JGI protein ID: 84348 | 1 | no |  |
|  | JGI protein ID: 86238 | 2 | no |  |
|  | JGI protein ID 87919 | 2 | no |  |
|  | JGI protein ID 55547 | 1 | no |  |
| *Cladosporium fulvum* | GenBank ID: 524933198 | 3 | no | Ecp6 |
| *Dothistroma septosporum* | GenBank: EME41175.1 | 3 | no |  |
| *Magnaporthe oryzae* | GenBank: EHA51101.1 | 2 | no | Slp1 |
| *Magnaporthe oryzae* | GenBank: EHA50138.1 | 2 | no | Slp2 |
| *Zymoseptoria tritici* | GenBank ID : XP_003848663.1 | 3 | no | Mg3LysM |
| *Zymoseptoria brevis* | GenBank: KJX93479.1 | 1 | no |  |
| *Colletotrichum higginsianum* | Broad Institute ID: CH063_13023 | 2 | no | ChELP1 |
| *Colletotrichum graminicola* | GenBank ID: EFQ27803.1 | 2 | no | CgELP2 |
| *Colletotrichum lindemuthianum* | GenBank ID : CAA04765.1 | 2 | no | ClCiH1 |

Table S1: List of LysM proteins used for BLAST analysis against *Clonostachys rosea* genome.

ChtBD1: Chitin binding domain
