# Supplementary material for: LysM Proteins Regulate Fungal Development and Contribute to Hyphal Protection and Biocontrol Traits in Clonostachys rosea
Source: Front Microbiol. 2020 Apr 16;11:679. doi: 10.3389/fmicb.2020.00679 (PMC7176902; doi:10.3389/fmicb.2020.00679)
Supplement: Supplementary file 3 [file Table_3.DOCX]

Table S3: List of primers used in this study

| Name | Target gene | Sequences (5´ → 3`) | Comment |
| --- | --- | --- | --- |
| 568 F | *lysm1* | ctggggatacctgctgggacat | Gene expression |
| 568 R |  | tcaatccgaggtgggcttctg |  |
| 6500 F | *lysm2* | gcacgaacctttggagcacag | Gene expression |
| 6500 R |  | tttcgcacggctgttgagttta |  |
| 568 ups F | *lysm1* upstream | ggggacaactttgtatagaaaagttgtggatgtcgactatgttggtgtg | Deletion cassette |
| 568 ups R |  | ggggactgcttttttgtacaaacttgtgtccgtttggtttagatgagtg |  |
| 568 ds F | *lysm1* down stream | ggggacagctttcttgtacaaagtggaagaagggctcgcatttgtgt | Deletion cassette |
| 568 ds R |  | ggggacaactttgtataataaagttggtgaaaagggctcgcagatag |  |
| 568 ko F | *lysm1* upstream | gttctttcaagatgcccgtagc | Mutant validation |
| 568 ko R |  | ctg cga agc ctc aaa gtt agt g |  |
| 6500 ups F | *lysm2* upstream | ggggacaactttgtatagaaaagttgcacccctcccacccgttgtat | Deletion cassette |
| 6500 ups R |  | ggggactgcttttttgtacaaacttgaaattgcccgttttggttcgc |  |
| 6500 ds F | *lysm2* down-stream | ggggacagctttcttgtacaaagtggtggaggacatggaggacgactg | Deletion cassette |
| 6500 ds R |  | ggggacaactttgtataataaagttgcgcatcaggcacgtagcagtc |  |
| 6500 ko F | *lysm2* upstream | ctg aat gcc cag cca cca aa | Mutant validation |
| 6500 ko R |  | tga cgc gat ctg gga agg aat |  |
| 568 comp R | *lysm1*  down-stream | ggggactgctttttttgtacaaacttgcaggcagtgctatggtgtctacg | Complementation cassette |
| 6500 comp R | *lysm2* down-stream | ggggactgctttttttgtacaaacttggaaaagttgatgtcgggaggag | Complementation cassette |
| Sur PCR F | *ILV1* | gggctatccccaccgagacct | Mutant validation |
| Sur PCR R |  | ggctacgcgtttgatggagtgct |  |
| Sur F | *ILV1* | ggggacaagtttgtacaaaaaagcaggctgtcgacgtgccaacgccacag | Selection cassette for chlorimuron ethyl |
| Sur R |  | ggggaccactttgtacaagaaagctgggtgtcgacgtgagagcatgcaat |  |
| Cr-actin F | Actin | gttctggattccggtgatggtgtc | Reference gene for RT-qPCR |
| Cr-actin R |  | tcggcagtggtggagaaggtgt |  |

^1^attB and attBr sequences for multisite gateway BP recombination are underlined;

Abbreviation used for enzyme encoding genes: *ILV1*: acetolactate synthase gene.
