# Supplementary material for: LysM Proteins Regulate Fungal Development and Contribute to Hyphal Protection and Biocontrol Traits in Clonostachys rosea
Source: Front Microbiol. 2020 Apr 16;11:679. doi: 10.3389/fmicb.2020.00679 (PMC7176902; doi:10.3389/fmicb.2020.00679)
Supplement: Supplementary file 4 [file Table_4.DOCX]

Table S4: GenBank accession number

| Species name | Gene name | GenBank ID |
| --- | --- | --- |
| *Clonostachys* *byssicola* | *lysm1* | MT037004 |
|  | *lysm2* | MT037005 |
|  | *chiC2* | MT037006 |
| *Conostachys rhizophaga* | *lysm1* | MT037007 |
|  | *lysm2* | MT037008 |
|  | *chiC2* | MT037009 |
| *Clonostachys chloroleuca* | *lysm1* | MT037010 |
|  | *lysm2* | MT037011 |
|  | *chiC2* | MT037012 |
| *Clonostachys solani* | *lysm1* | MT037013 |
|  | *lysm2* | MT037014 |
|  | *chiC2* | MT037015 |
