# Supplementary material for: LysM Proteins Regulate Fungal Development and Contribute to Hyphal Protection and Biocontrol Traits in Clonostachys rosea
Source: Front Microbiol. 2020 Apr 16;11:679. doi: 10.3389/fmicb.2020.00679 (PMC7176902; doi:10.3389/fmicb.2020.00679)
Supplement: Supplementary file 5 [file Data_Sheet_1.PDF]

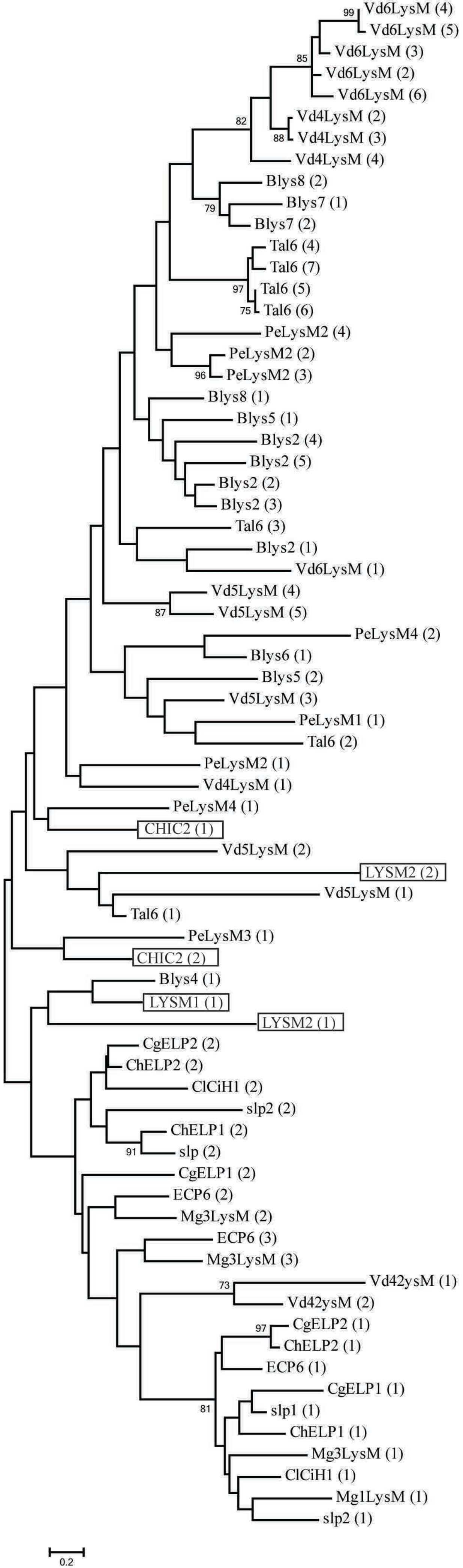
Vd6LysM (4)
Vd6LysM (5)
Vd6LysM (3)
Vd6LysM (2)
Vd6LysM (6)
Vd4LysM (2)
Vd4LysM (3)
Vd4LysM (4)
Blys8 (2)
Blys7 (1)
Blys7 (2)
Tal6 (4)
Tal6 (7)
Tal6 (5)
Tal6 (6)
PeLysM2 (4)
PeLysM2 (2)
PeLysM2 (3)
Blys8 (1)
Blys5 (1)
Blys2 (4)
Blys2 (5)
Blys2 (2)
Blys2 (3)
Tal6 (3)
Blys2 (1)
Vd6LysM (1)
Vd5LysM (4)
Vd5LysM (5)
PeLysM4 (2)
Blys6 (1)
Blys5 (2)
Vd5LysM (3)
PeLysM1 (1)
Tal6 (2)
PeLysM2 (1)
Vd4LysM (1)
PeLysM4 (1)
CHIC2 (1)
Vd5LysM (2)
LYSM2 (2)
Vd5LysM (1)
Tal6 (1)
PeLysM3 (1)
CHIC2 (2)
Blys4 (1)
LYSM1 (1)
LYSM2 (1)
CgELP2 (2)
ChELP2 (2)
ClCiH1 (2)
slp2 (2)
ChELP1 (2)
slp (2)
CgELP1 (2)
ECP6 (2)
Mg3LysM (2)
ECP6 (3)
Mg3LysM (3)
Vd42ysM (1)
Vd42ysM (2)
CgELP2 (1)
ChELP2 (1)
ECP6 (1)
CgELP1 (1)
slp1 (1)
ChELP1 (1)
Mg3LysM (1)
ClCiH1 (1)
Mg1LysM (1)
slp2 (1)
99
85
82
88
79
97
75
96
87
91
73
97
81
0.2

Figure S1: Phylogenetic analysis of LysM modules. LysM module protein sequences were aligned using Muscle (Edgar, 2004) and phylogenetic analysis was performed using maximum likelihood methods implemented in MEGA ver. 6 (Tamura et al., 2013). Statistical support for branches was assessed by 1000-iteration bootstrap resampling. Branch support values (bootstrap proportions ≥ 70%) are associated with nodes. The LysM modules from *Clonostachys rosea* are highlighted in rectangle. The number in parenthesis indicates the position of LysM module within the LysM protein.

Accession number of proteins: *Cladosporium Fulvum* Ecp6: PDB: 4B9H_A; *Magnaporthe oryzae* slp1: EHA51101.1; *Magnaporthe oryzae* slp2: EHA50138.1; *Mycosphaerella graminicola* Mg3LysM: XP_003850050.1; *Mycosphaerella graminicola* Mg1LysM (Marshall et al., 2011); *Colletotrichum higginsianum* ChELP1 and ChELP2 (Takahara et al., 2016); *Colletotrichum graminicola* CgELP1: EFQ32497.1; *Colletotrichum graminicola* CgELP2: EFQ27803.1; *Colletotrichum lindemuthianum* ClCiH1: CAA04765.1; *Trichoderma atroviride* Tal6: XP_013947368.1; *Verticillium dahliae* Vd2LysM: XP_009653211.1; *Verticillium dahliae* Vd4LysM: XP_009648083.1; *Verticillium dahliae* Vd5LysM: XP_009652680.1; *Verticillium dahliae* Vd6LysM: XP_009654198.1; *Beauveria bassiana* Blys2: XP_008596457.1; *Beauveria bassiana* Blys4: XP_008599172.1; *Beauveria bassiana* Blys5: XP_008601921.1; *Beauveria bassiana* Blys6: XP_008602072.1; *Beauveria bassiana* Blys7: XP_008602298.1; *Beauveria bassiana* Blys8: XP_008602669.1; *Penicillium expansum* PeLysM1: XP_016599271.1; *Penicillium expansum* PeLysM2: XP_016600130.1; *Penicillium expansum* PeLysM3: XP_016600884.1; *Penicillium expansum* PeLysM4: XP_016594789.1; *Clonostachys rosea* LYSM1: MT037001; *Clonostachys rosea* LYSM2: MT037002; *Clonostachys rosea* CHIC2: MT037003.

Takahara H, Hacquard S, Kombrink A, Hughes HB, Halder V, Robin GP, et al. *Colletotrichum higginsianum* extracellular LysM proteins play dual roles in appressorial function and suppression of chitin-triggered plant immunity. New Phytol. 2016; 211:1323-1337.

Marshall R, Kombrink A, Motteram J, Loza-Reyes E, Lucas J, Hammond-Kosack KE, et al. Analysis of two in planta expressed LysM effector homologs from the fungus *Mycosphaerella graminicola* reveals novel functional properties and varying contributions to virulence on wheat. Plant Physiol. 2011; 156:756-769.
