# Supplementary material for: LysM Proteins Regulate Fungal Development and Contribute to Hyphal Protection and Biocontrol Traits in Clonostachys rosea
Source: Front Microbiol. 2020 Apr 16;11:679. doi: 10.3389/fmicb.2020.00679 (PMC7176902; doi:10.3389/fmicb.2020.00679)
Supplement: Supplementary file 6 [file Data_Sheet_2.PDF]

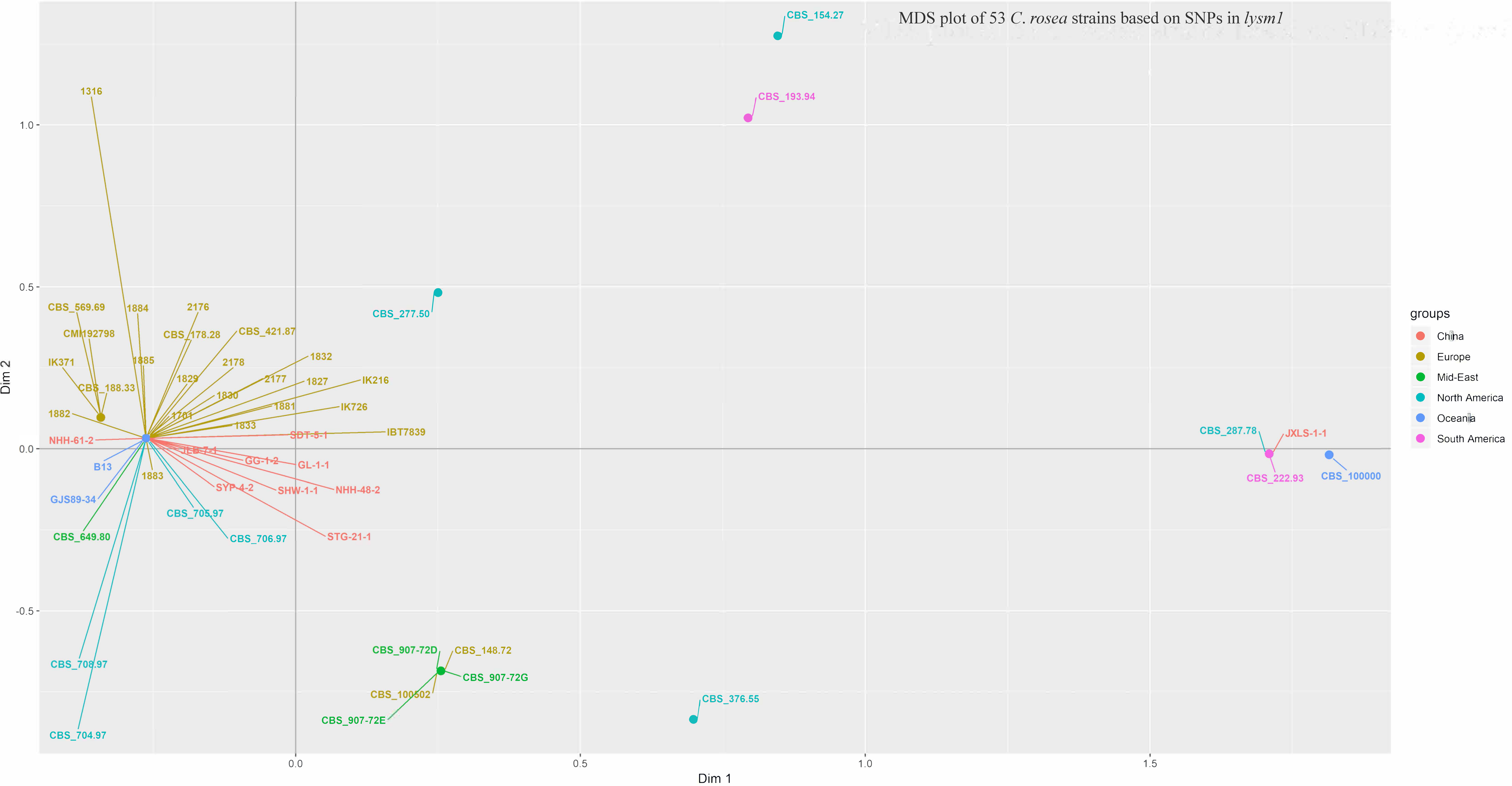

MDS plot of 53 C. rosea strains based on SNPs in lysm1
Dim 1
Dim 2
groups
China
Europe
Mid-East
North America
Oceania
South America
CBS_154.27
CBS_193.94
1316
CBS_277.50
CBS_569.69
1884
2176
CMI192798
CBS_178.28
CBS_421.87
IK371
1885
2178
1832
1829
2177
1827
IK216
CBS_188.33
1830
1881
IK726
1882
1701
1833
IBT7839
NHH-61-2
SDT-5-1
JLB-7-1
GG-1-2
GL-1-1
B13
1883
SYP-4-2
SHW-1-1
NHH-48-2
GJS89-34
CBS_705.97
CBS_649.80
CBS_706.97
STG-21-1
CBS_287.78
JXLS-1-1
CBS_222.93
CBS_100000
CBS_708.97
CBS_704.97
CBS_907-72D
CBS_148.72
CBS_907-72G
CBS_100502
CBS_907-72E
CBS_376.55

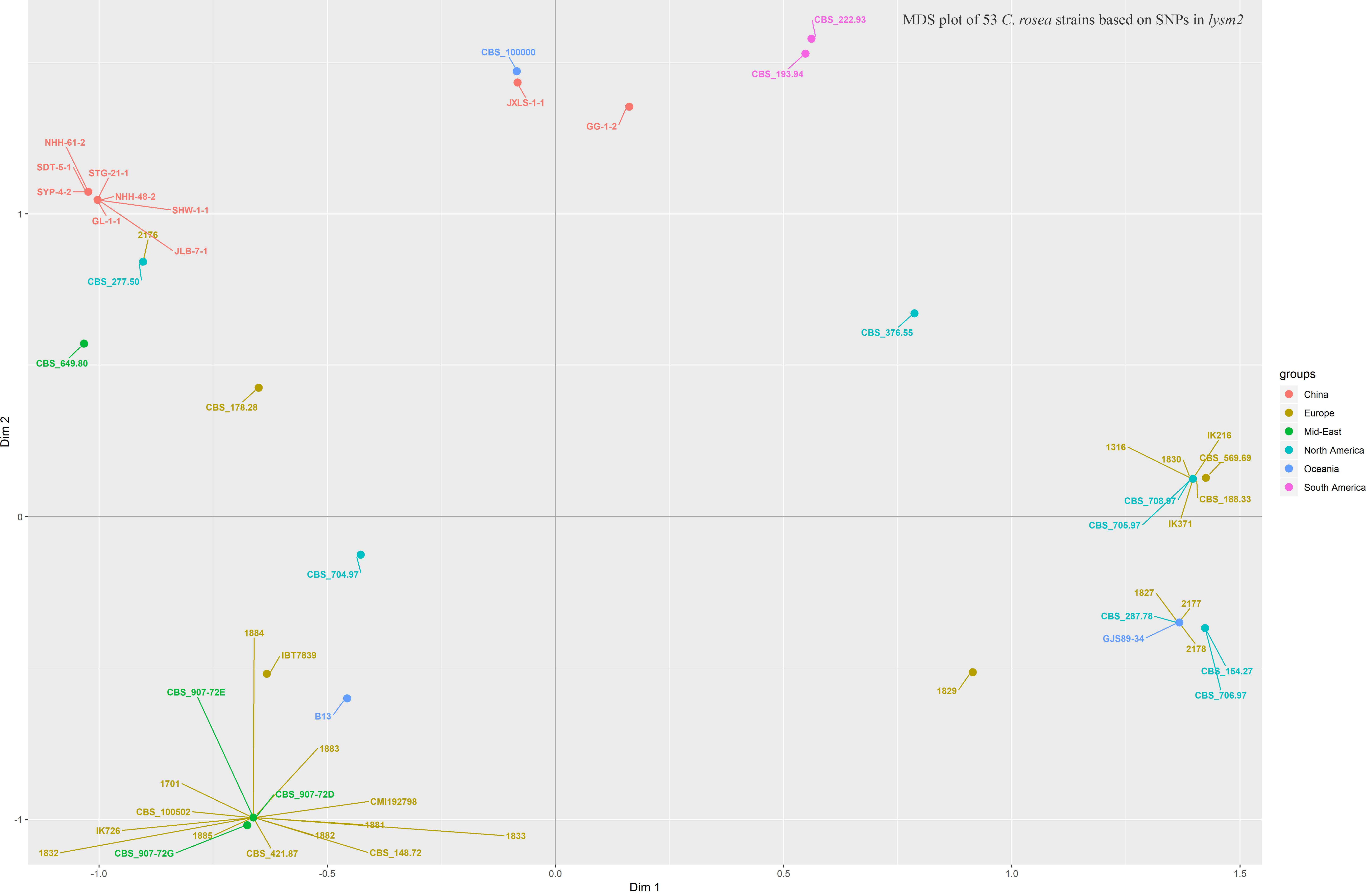
MDS plot of 53 C. rosea strains based on SNPs in lysm2
Dim 1
Dim 2
groups
China
Europe
Mid-East
North America
Oceania
South America
CBS_222.93
CBS_193.94
CBS_100000
JXLS-1-1
GG-1-2
NHH-61-2
SDT-5-1
STG-21-1
SYP-4-2
NHH-48-2
SHW-1-1
GL-1-1
2176
JLB-7-1
CBS_277.50
CBS_376.55
CBS_649.80
CBS_178.28
IK216
1316
1830
CBS_569.69
CBS_708.97
CBS_188.33
CBS_705.97
IK371
CBS_704.97
1827
2177
CBS_287.78
GJS89-34
2178
CBS_154.27
CBS_706.97
1829
1884
IBT7839
CBS_907-72E
B13
1883
1701
CBS_907-72D
CMI192798
CBS_100502
1881
IK726
1885
1882
1833
1832
CBS_907-72G
CBS_421.87
CBS_148.72
-1.0
-0.5
0.0
0.5
1.0
1.5
1
0
-1

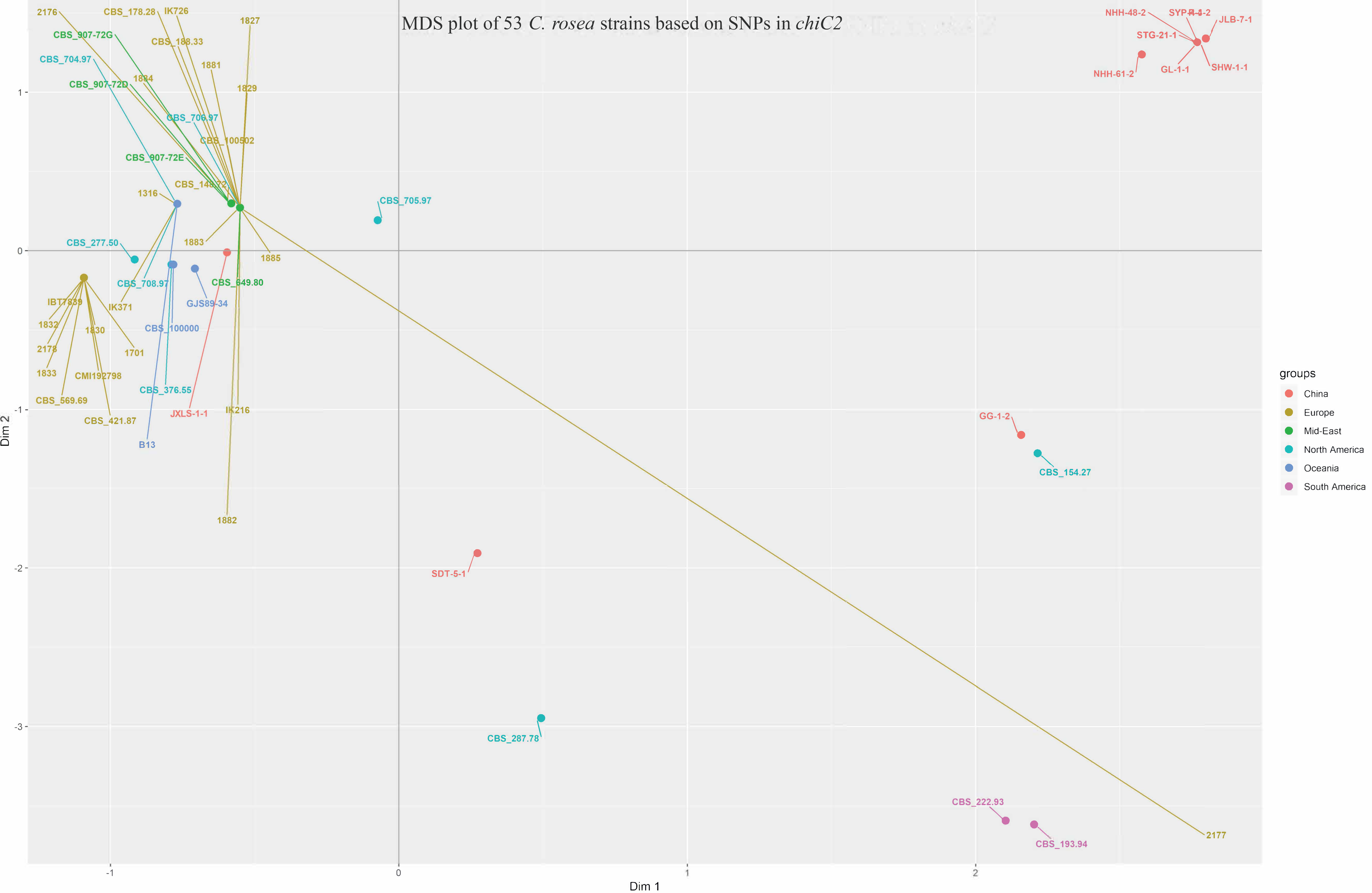

MDS plot of 53 C. rosea strains based on SNPs in chiC2
Dim 1
Dim 2
groups
China
Europe
Mid-East
North America
Oceania
South America
2176
CBS_178.28
IK726
1827
CBS_907-72G
CBS_188.33
CBS_704.97
1881
1884
CBS_907-72D
1829
CBS_706.97
CBS_100502
CBS_907-72E
CBS_148.72
1316
CBS_705.97
CBS_277.50
1883
1885
CBS_708.97
CBS_649.80
IBT7839
IK371
GJS89-34
1832
1830
CBS_100000
2178
1701
1833
CMI192798
CBS_376.55
CBS_569.69
JXLS-1-1
IK216
CBS_421.87
B13
1882
NHH-48-2
STG-21-1
JLB-7-1
NHH-61-2
GL-1-1
SHW-1-1
GG-1-2
CBS_154.27
SDT-5-1
CBS_287.78
CBS_222.93
CBS_193.94
2177

Figure S2: Multidimensional scaling (MDS) plots of SNP genotypes from a world-wide collection of 53 *Clonostachys rosea* strains in *lysm1*, *lysm2* and *chiC2*.

A multi-dimensional scaling (MDS) analysis of the distribution of *lysm1* SNPs, did not indicate any correlation between sequence variation in *lysm1* and the geographical origin of the strains (Fig S2). The 3'-end of the *lysm1* alleles from three strains (CBS 10000, CBS 222.93 and CBS 287.78) were different from the other 50 strains in that the last 177 bp (from pos. 355) were replaced by a different 117 bp DNA fragment. This effectively removed the LysM module from these alleles, while preserving the trans-membrane domain. After removing the 117 bp DNA fragment from the sequence alignment, a test for the presence of recombination using GARD indicated ($P < 0.001$) one potential recombination event at position 192. Furthermore, analysis with the codon-based maximum likelihood method REL provided no evidence for positive selection in *lysm1*.

MDS analysis of sequence polymorphism in *lysm2* showed that strains originating from China, except GG-1-2 and JXLS-1-1, grouped together, while European strains were found in two groups where one group clustered together with strains originating from North America while second group clustered with strains from the Middle East (Fig S2). REL analysis indicated one site evolving under positive selection (Bayes factor = 46), at codons 205 (Fig. 1).

Based on a MDS analysis of sequence polymorphism in *chiC2*, strains from China grouped together, with the exception for JXLS-1-1, SDT-5-1 and GG-1-2. Strains originating from Europe formed two groups where one group only contained European strains, while the other group included strains with a wide geographical distribution (Fig S2). GARD analysis indicated ($P < 0.001$) one potential recombination event at position 531. REL analysis indicated one site to evolve under positive selection (Bayes factor = 699), at codon 826 in the GH18 module (Fig. 1).
