# Supplementary material for: LysM Proteins Regulate Fungal Development and Contribute to Hyphal Protection and Biocontrol Traits in Clonostachys rosea
Source: Front Microbiol. 2020 Apr 16;11:679. doi: 10.3389/fmicb.2020.00679 (PMC7176902; doi:10.3389/fmicb.2020.00679)
Supplement: Supplementary file 7 [file Data_Sheet_3.PDF]

Figure S3

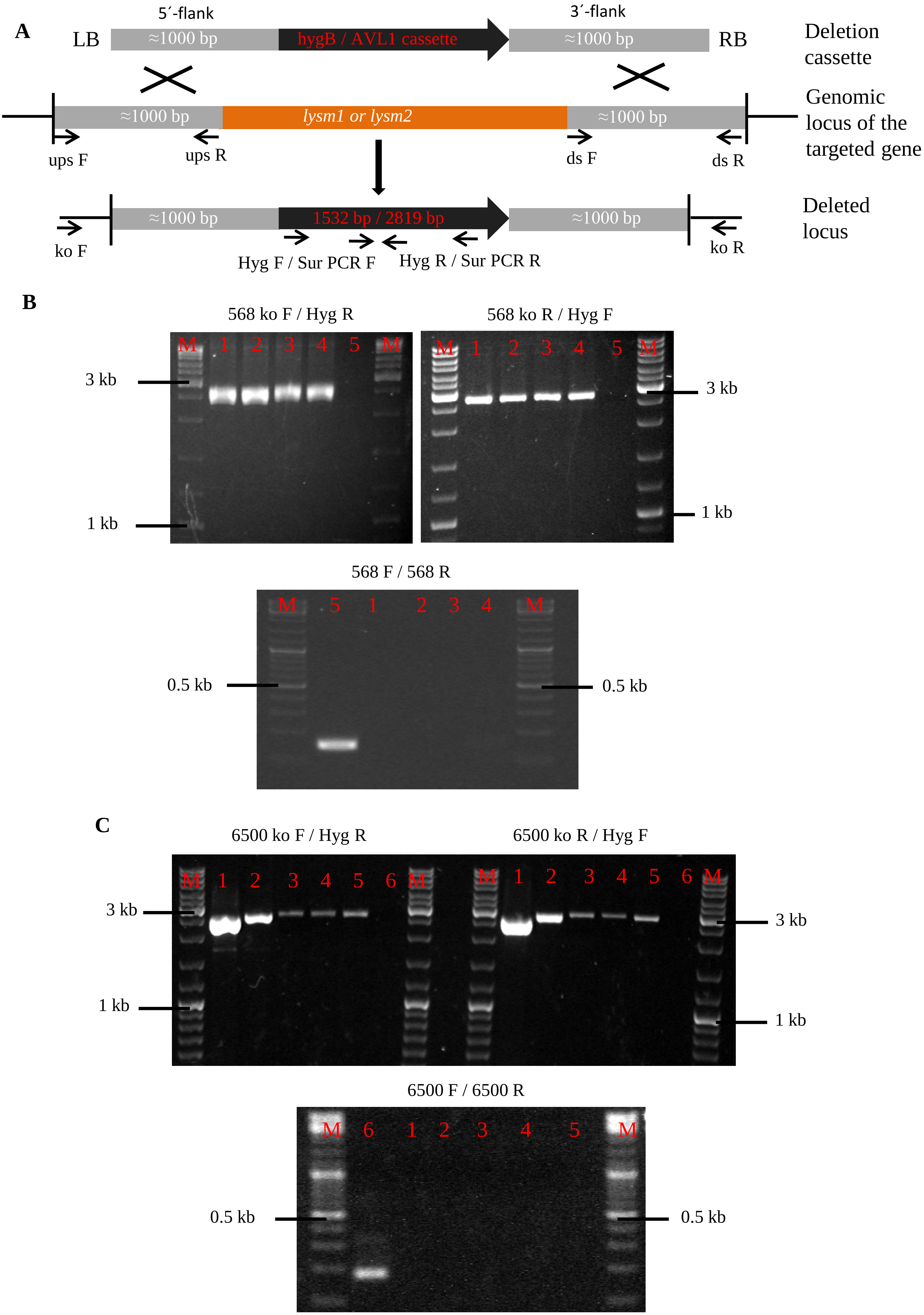

A
5´-flank
3´-flank
LB
≈1000 bp
hygB / AVL1 cassette
≈1000 bp
RB
Deletion cassette
≈1000 bp
lysm1 or lysm2
≈1000 bp
Genomic locus of the targeted gene
ups F
ups R
ds F
ds R
≈1000 bp
1532 bp / 2819 bp
≈1000 bp
Deleted locus
ko F
Hyg F / Sur PCR F
Hyg R / Sur PCR R
ko R
B
568 ko F / Hyg R
568 ko R / Hyg F
M 1 2 3 4 5 M
M 1 2 3 4 5 M
3 kb
1 kb
3 kb
1 kb
568 F / 568 R
M 5 1 2 3 4 M
0.5 kb
0.5 kb
C
6500 ko F / Hyg R
6500 ko R / Hyg F
M 1 2 3 4 5 6 M
M 1 2 3 4 5 6 M
3 kb
1 kb
3 kb
1 kb
6500 F / 6500 R
M 6 1 2 3 4 5 M
0.5 kb
0.5 kb

D

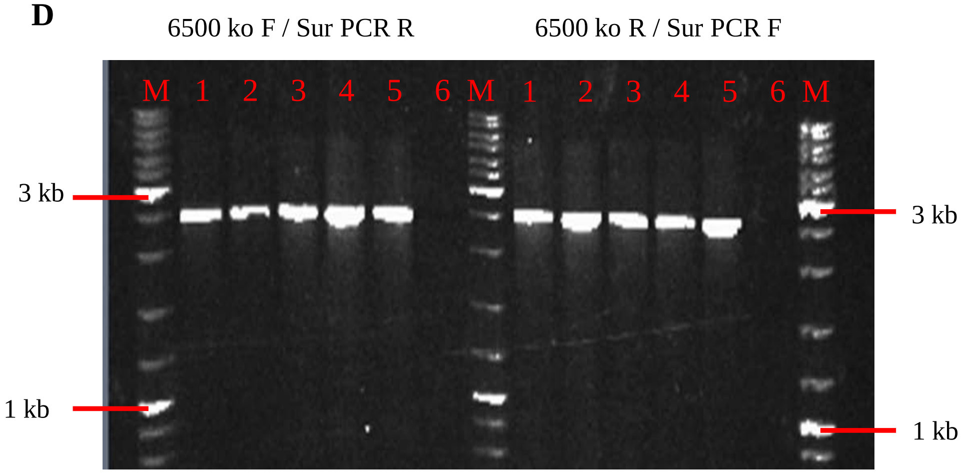
6500 ko F / Sur PCR R
6500 ko R / Sur PCR F
M 1 2 3 4 5 6 M 1 2 3 4 5 6 M
3 kb
1 kb
3 kb
1 kb

E

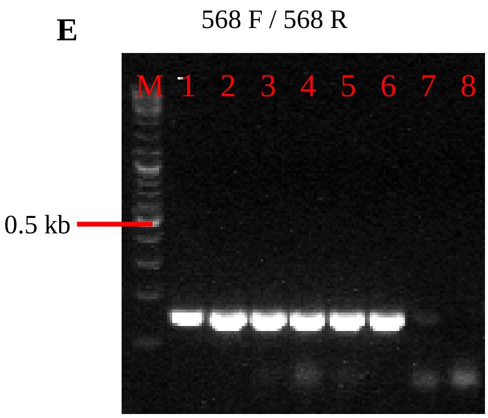
568 F / 568 R
M 1 2 3 4 5 6 7 8
0.5 kb

F

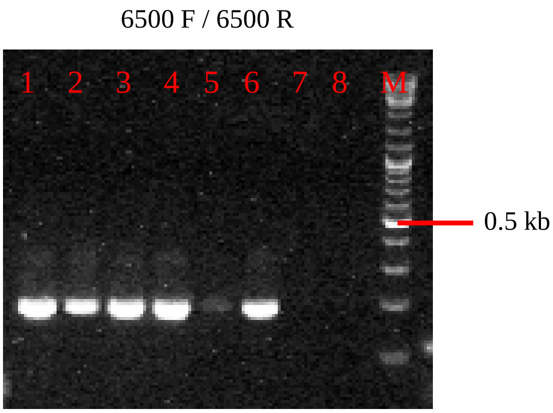
6500 F / 6500 R
1 2 3 4 5 6 7 8 M
0.5 kb

Figure S3: Schematic representation of deletion cassettes, and validation of mutant strains using PCR and RT-PCR.

A: Organisation of *lysm1 or lysm2* locus in WT and mutant strains of *C. rosea*. The coding region of respective gene was replaced by hygB (or ILV1 for double deletion) cassette by homologous recombination resulting in generation of deletion strains. The arrow heads indicate the location of primers used to construct the deletion cassette and analysis of mutants using PCR. Abbreviations: LB, left border; RB, right border.

B: PCR verification of Δ*lysm1* strains using primers located in the hygB cassette (Hyg F / Hyg R) in combination with primers located upstream and downstream from the deletion cassette (568 ko F / 568 ko R). A PCR product of ~2.7 kb using primers 568 ko F / Hyg R, and 568 ko R / Hyg F were expected from a correct gene replacement. RT-PCR analysis of *lysm1* gene expression in WT and deletion strains using *lysm1* specific primers 568 F / 568 R. A PCR product of 140 bp was expected from WT. M, gene ruler DNA ladder mix; 1-4, independent Δ*lysm1* mutants; 5, WT strain.

C: PCR verification of Δ*lysm2* using primers located in the hygB cassette (Hyg F / Hyg R) in combination with primers located upstream and downstream from the deletion cassette (6500 ko F / 6500 ko R). A PCR product of ~2.7 kb using primers 6500 ko F / Hyg R, and 6500 ko R / Hyg F were expected from a correct gene replacement. RT-PCR analysis of *lysm2* gene expression in WT and deletion strains using *lysm2* specific primers. A PCR product of 173 bp was expected from WT. M, gene ruler DNA ladder mix; 1-5, independent Δ*lysm2* mutants; 6, WT strain.

D: PCR verification of Δ*lysm1*Δ*lysm2* using primers located in the ILV1 cassette (Sur PCR F / Sur PCR R) in combination with primers located upstream and downstream from the deletion cassette (6500 ko F / 6500 ko R). A PCR product of ~2.7 kb using primers 6500 ko F / Sur PCR R, and 6500 ko R / Sur PCR F were expected from a correct gene replacement.

E: Validation of complementation strains. RT-PCR analysis of *lysm1* expression in WT, Δ*lysm1*, and Δ*lysm1*+ complemented strains, using *lysm1* specific 568 F/568 R primers. M, gene ruler DNA ladder mix; 1-5, independent Δ*lysm1*+ strains; 6, WT; 7, Δ*lysm1*; 8, water control.

F: Validation of complementation strains. RT-PCR analysis of *lysm2* expression in WT, Δ*lysm2*, and Δ*lysm1*+ complemented strains, using *lysm2* specific 6500 F/6500 R primers. M, gene ruler DNA ladder mix; 1-5, independent Δ*lysm2*+ strains; 6, WT; 7, Δ*lysm2*; 8, water control.

Primer combinations used for PCR and RT-PCR are given above the images.
