# Supplementary material for: LysM Proteins Regulate Fungal Development and Contribute to Hyphal Protection and Biocontrol Traits in Clonostachys rosea
Source: Front Microbiol. 2020 Apr 16;11:679. doi: 10.3389/fmicb.2020.00679 (PMC7176902; doi:10.3389/fmicb.2020.00679)
Supplement: Supplementary file 8 [file Data_Sheet_4.PDF]

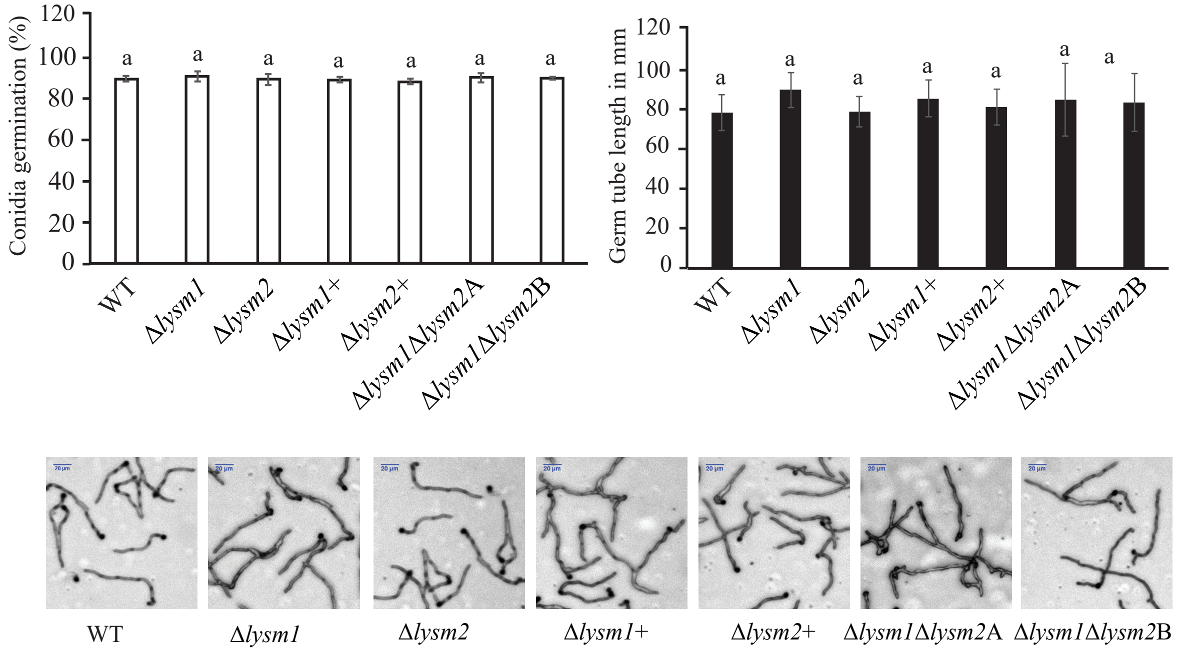

Figure S4: Analysis of conidial germination and germ tube length 24 h post inoculation. Conidia from *C. rose*a WT and the deletion strains were inoculated on half-strength PDA medium on microscope slides and incubated at 25 °C for 24 hour. Photographs were taken in a Leica DM5500M Microscope at 20 X magnification using a Leica DFC360FX digital camera equipped with the Microscope (Wetzlar, Germany). Frequency of germinating conidia was determined by counting the number of germinating and non-germinating conidia, while length of the germ was measured using ImageJ software. Scale bar: 20 μm. Error bars represent standard deviation based on four biological replicates. Different letters indicate statistically significant differences ($P \leq 0.05$) within the experiments based on Fisher's exact test.
