# Supplementary material for: LysM Proteins Regulate Fungal Development and Contribute to Hyphal Protection and Biocontrol Traits in Clonostachys rosea
Source: Front Microbiol. 2020 Apr 16;11:679. doi: 10.3389/fmicb.2020.00679 (PMC7176902; doi:10.3389/fmicb.2020.00679)
Supplement: Supplementary file 9 [file Data_Sheet_5.PDF]

Figure S5

Phenotypic characterizations of *C. rosea* WT and LysM deletion mutants. Agar plugs of *C. rosea* strains were inoculated on in 5 cm diameter PDA plates and incubated at 25°C. Plates were monitored weekly and photographs were taken by scanning the plate using Epson Perfection V700 Photo (Epson, Suwa, Japan). Pixel intensity of the colony was measured using ImageJ and used as a proxy for mycelial biomass. Error bars represent standard deviation based on three biological replicates. Different letters indicate statistically significant differences ($P \leq 0.05$) within the experiments based on Fisher's exact test.

WT, wild type; lysm2 ko, *lysm2* deletion strain Δ*lysm2*; lysm1 ko; *lysm1* deletion strain Δ*lysm1*, lysm2+, *lysm2* complementation strain; lysm1+, *lysm1* complementation strain; lysm1lysm2 ko; *lysm1*, *lysm2* double deletion strain Δ*lysm1*Δ*lysm2*.

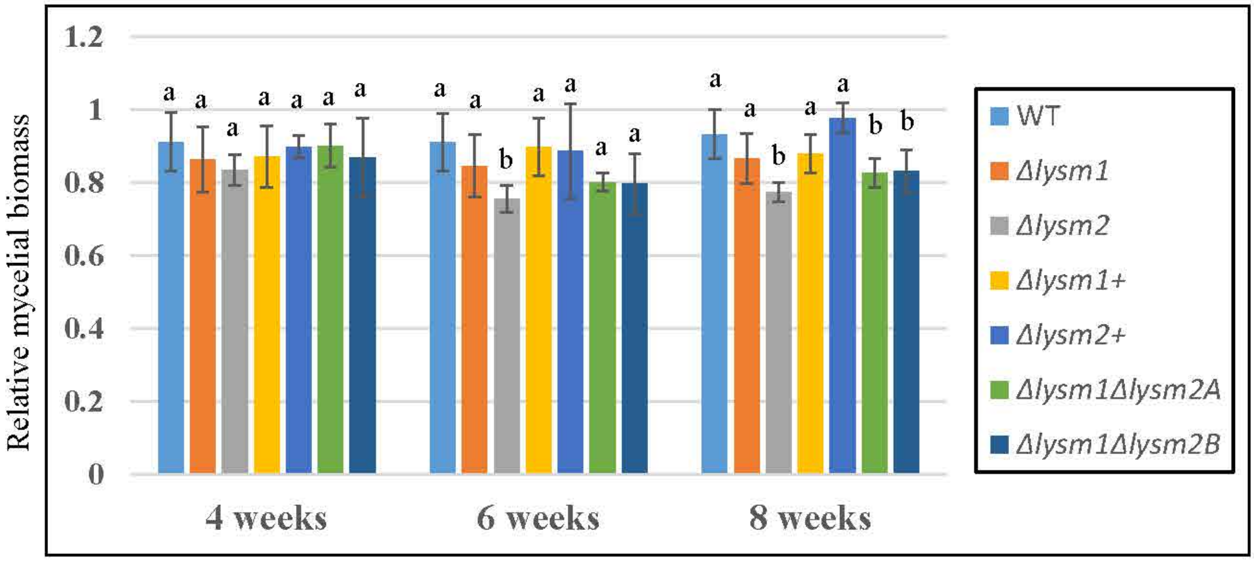

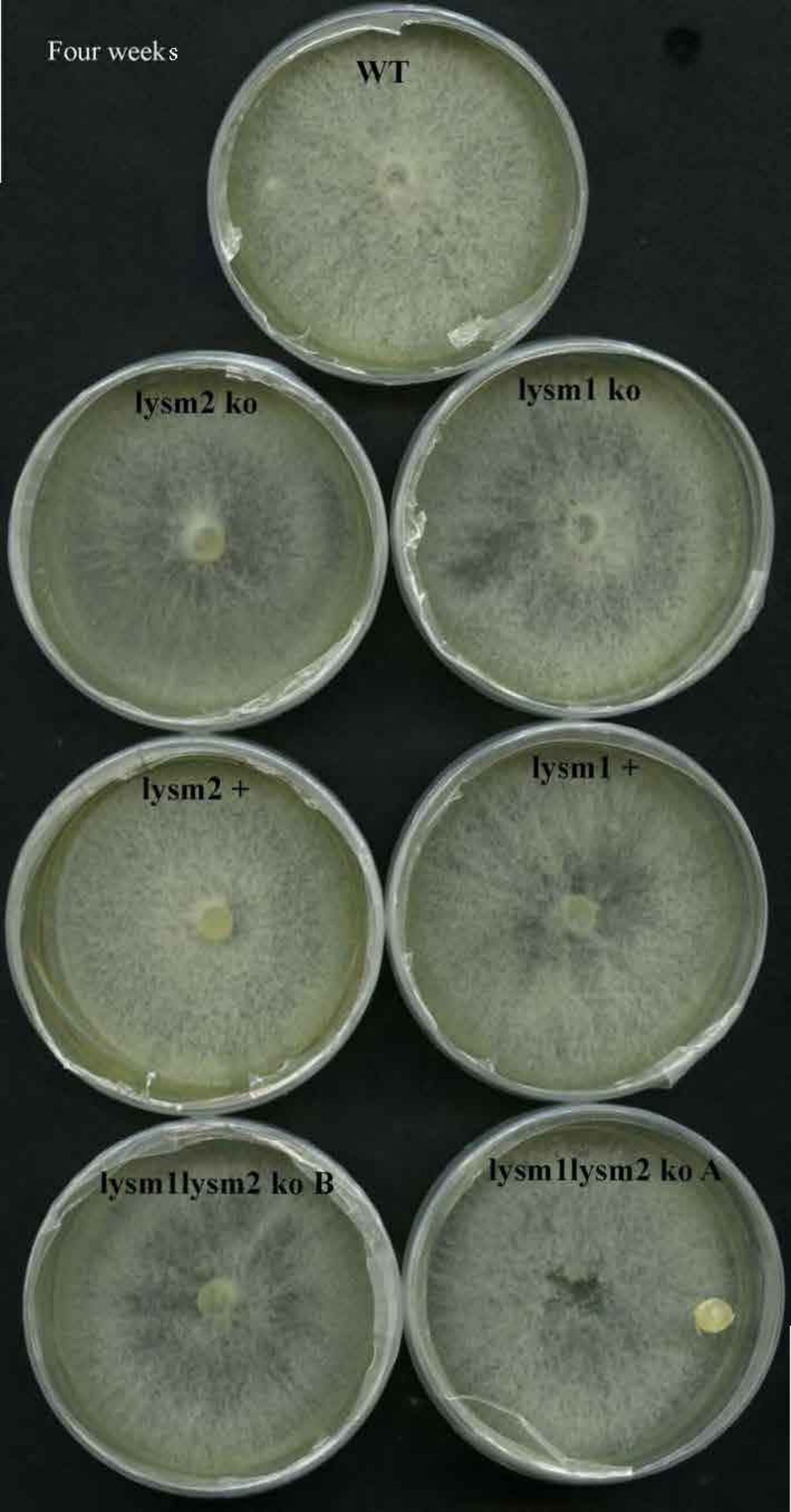
Four weeks
WT
lysm2 ko
lysm1 ko
lysm2 +
lysm1 +
lysm1lysm2 ko B
lysm1lysm2 ko A

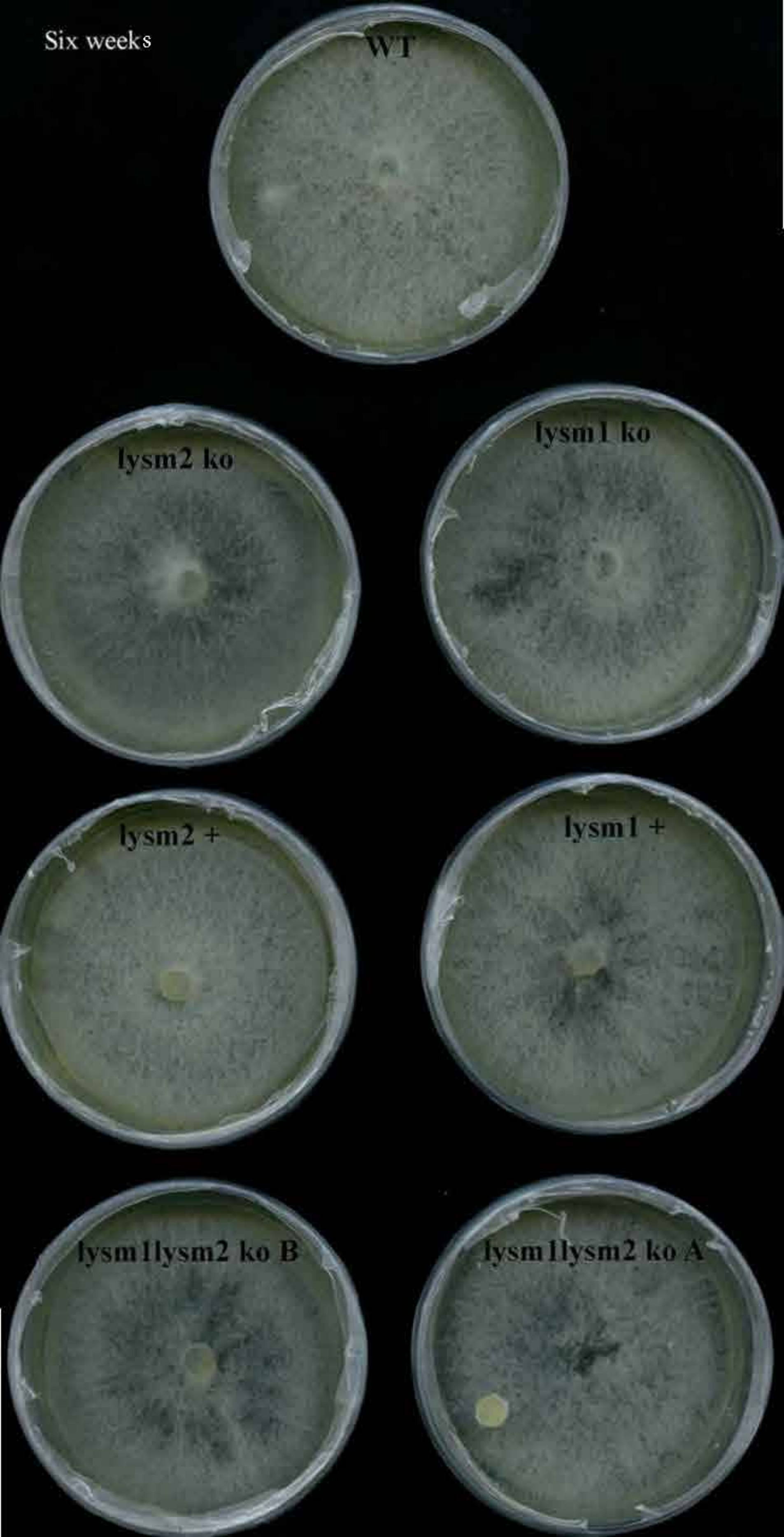

Six weeks
WT
lysm2 ko
lysm1 ko
lysm2 +
lysm1 +
lysm1lysm2 ko B
lysm1lysm2 ko A

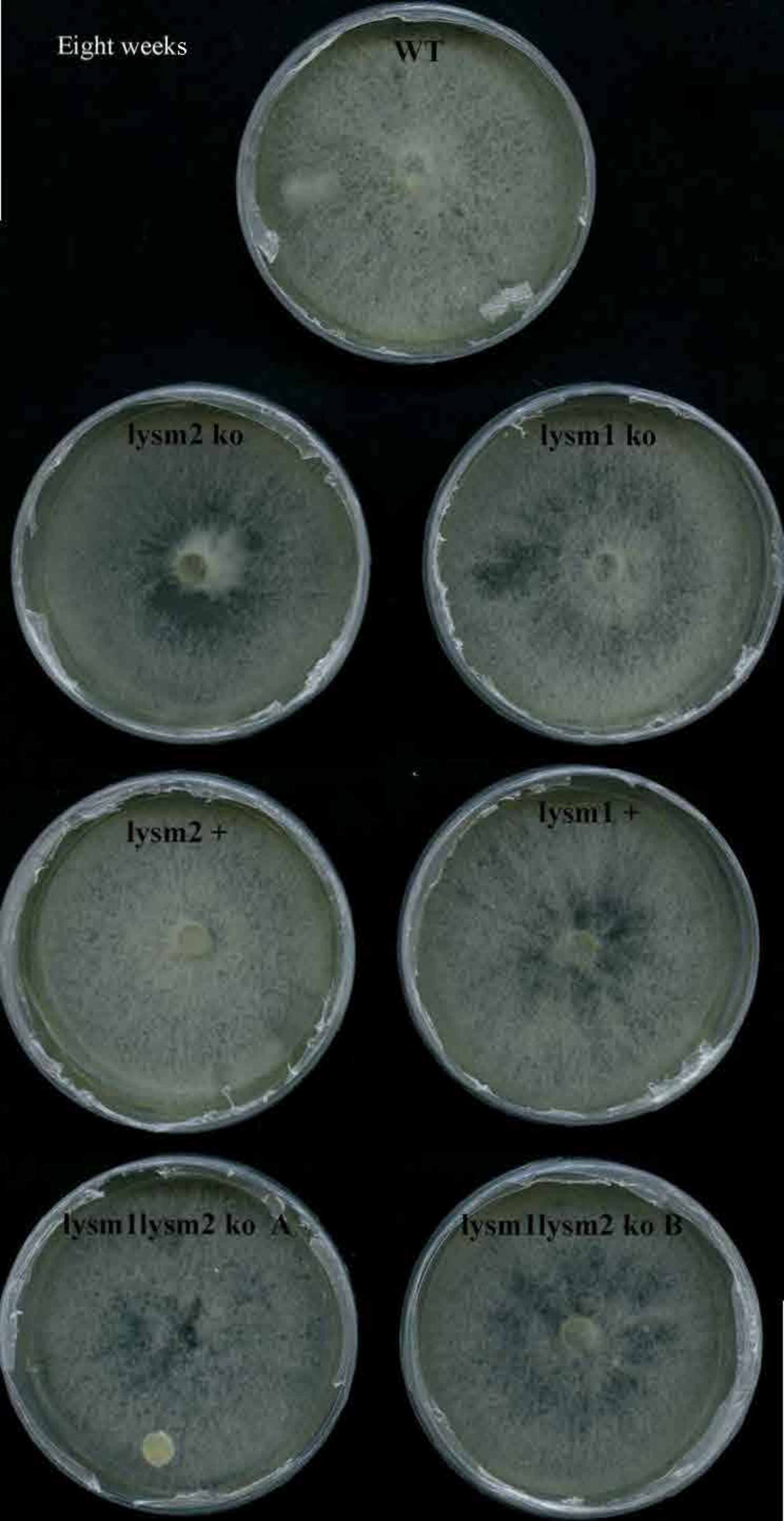
Eight weeks
WT
lysm2 ko
lysm1 ko
lysm2 +
lysm1 +
lysm1lysm2 ko A
lysm1lysm2 ko B
