# Supplementary material for: LysM Proteins Regulate Fungal Development and Contribute to Hyphal Protection and Biocontrol Traits in Clonostachys rosea
Source: Front Microbiol. 2020 Apr 16;11:679. doi: 10.3389/fmicb.2020.00679 (PMC7176902; doi:10.3389/fmicb.2020.00679)
Supplement: Supplementary file 10 [file Data_Sheet_6.pdf]

Figure S6

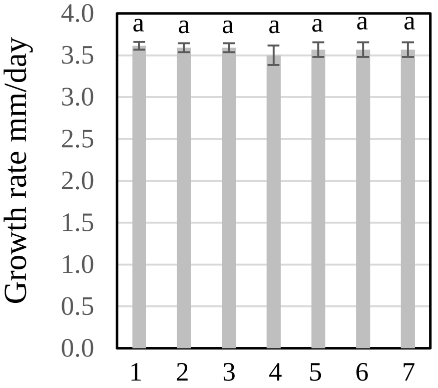

Growth rate of *C. rosea* WT and deletion strains in dual culture assay against *B. cinerea* or *F. graminearum*

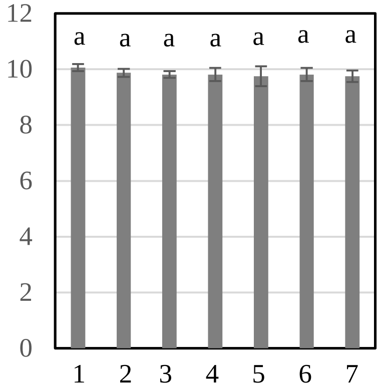

Growth rate of *B. cinerea* in dual culture assay against *C. rosea* WT and deletion strains

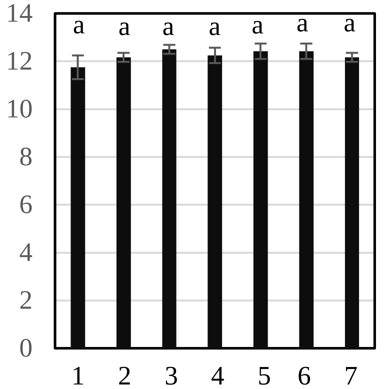

Growth rate of *F. graminearum* in dual culture assay against *C. rosea* WT and deletion strains

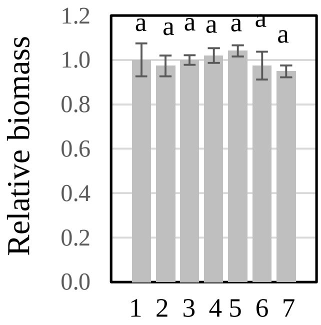

Relative biomass of *C. rosea* strains in dual culture assay against *B. cinerea* at mycelial contact stage

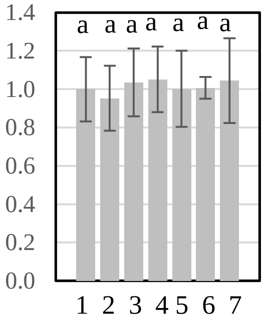

Relative biomass of *C. rosea* strains in dual culture assay against *F. graminearum* at mycelial contact stage

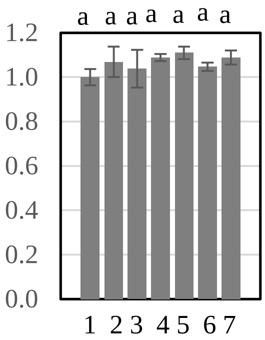

Relative biomass of *B. cinerea* in dual culture assay against *C.* rosea strains at mycelial contact stage

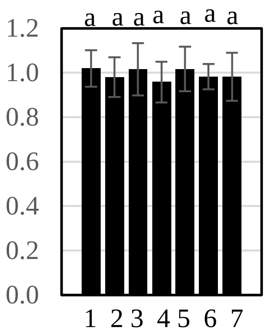

Relative biomass of *F. graminearum* in dual culture assay against *C. rosea* strains at mycelial contact stage

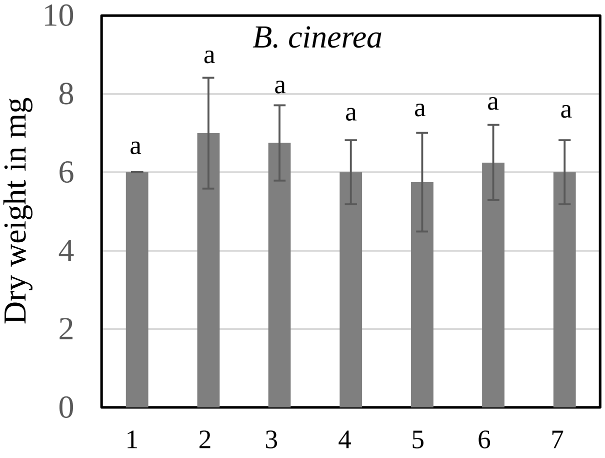

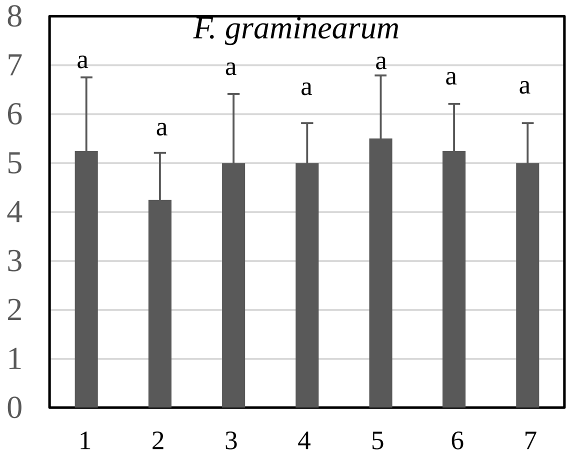

Biomass (mycelial dry weight in mg) of *C. rosea* WT and deletion strains in culture filtrates from *B. cinerea* or *F. graminearum* four days post inoculations.

Figure S6: Dual culture assay to analyse antagonistic interactions against *B. cinerea* and *F. graminearum*. Agar plugs of *C. rosea* strains were inoculated on opposite sides in nine cm diameter agar plates and incubated at 25°C. After seven days of incubation, a plug of *B. cinerea* or *F. graminearum* was placed at equal distance to the opposite edge of the plate.

Growth rate of *C. rosea* strains, *B. cinerea* or *F. graminearum* was recorded daily till the mycelial contact. Photographs of dual culture assay were taken at four days post inoculation of *B. cinerea* or *F. graminearum;* or 11 days post inoculation of *C. rosea* strains (at this time point mycelial front of *C. rosea* strains touched the mycelial front of *B. cinerea* or *F. graminearum*); and after 14 days inoculation of *B. cinerea* or *F. graminearum;* or 21 days post inoculation of *C. rosea* strains (at this time point *C. rosea* mycelium overgrow *B. cinerea* and reached to opposite wall of the Petri dish).

Pixel intensity of the images was measured with ImageJ image analysis software [70] and normalized to the value of the WT. Pixel intensity was used as a proxy to quantify the mycelial biomass.

For culture filtrate test, culture filtrates from WT and deletion strains grown in PDB were collected 10 days post-inoculation and then inoculated with a *B. cinerea* or *F. graminearum* agar plug. Biomass production in culture filtrates was analysed by determining mycelial dry weight four days post-inoculation.

Error bars represent standard deviation based on 4 biological replicates.
The experiments were carried out in four biological replicates. Same letters indicate no statistically significant differences ($P \leq 0.05$) within experiments based on the Fisher test.

Abbreviations1: *C. rosea* WT, 2: Δ*lysm1*, 3: Δ*lysm2*, 4: Δ*lysm1+*, 5: Δ*lysm2+*, 6: Δ*lysm1*Δ*lysm2*A, 7: Δ*lysm1*Δ*lysm2*B.

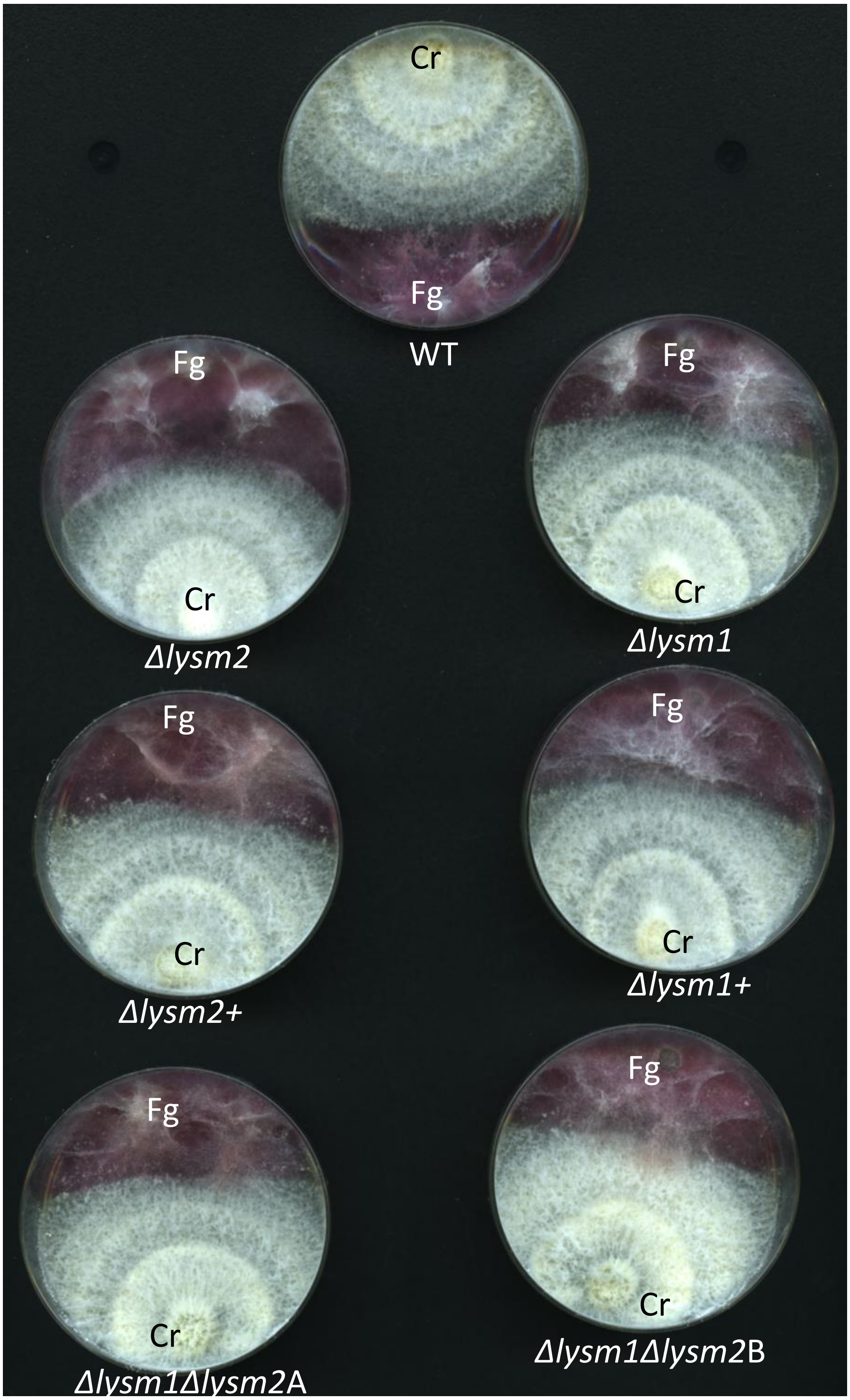

Figure S6: Mycoparasitism analysis of *C. rosea* WT and deletion strains in five cm diameter plate against *F. graminearum*. The experiment was performed in five biological replicates and photographs of representative plates were taken three weeks post inoculation.
