# Supplementary material for: LysM Proteins Regulate Fungal Development and Contribute to Hyphal Protection and Biocontrol Traits in Clonostachys rosea
Source: Front Microbiol. 2020 Apr 16;11:679. doi: 10.3389/fmicb.2020.00679 (PMC7176902; doi:10.3389/fmicb.2020.00679)
Supplement: Supplementary file 11 [file Data_Sheet_7.PDF]

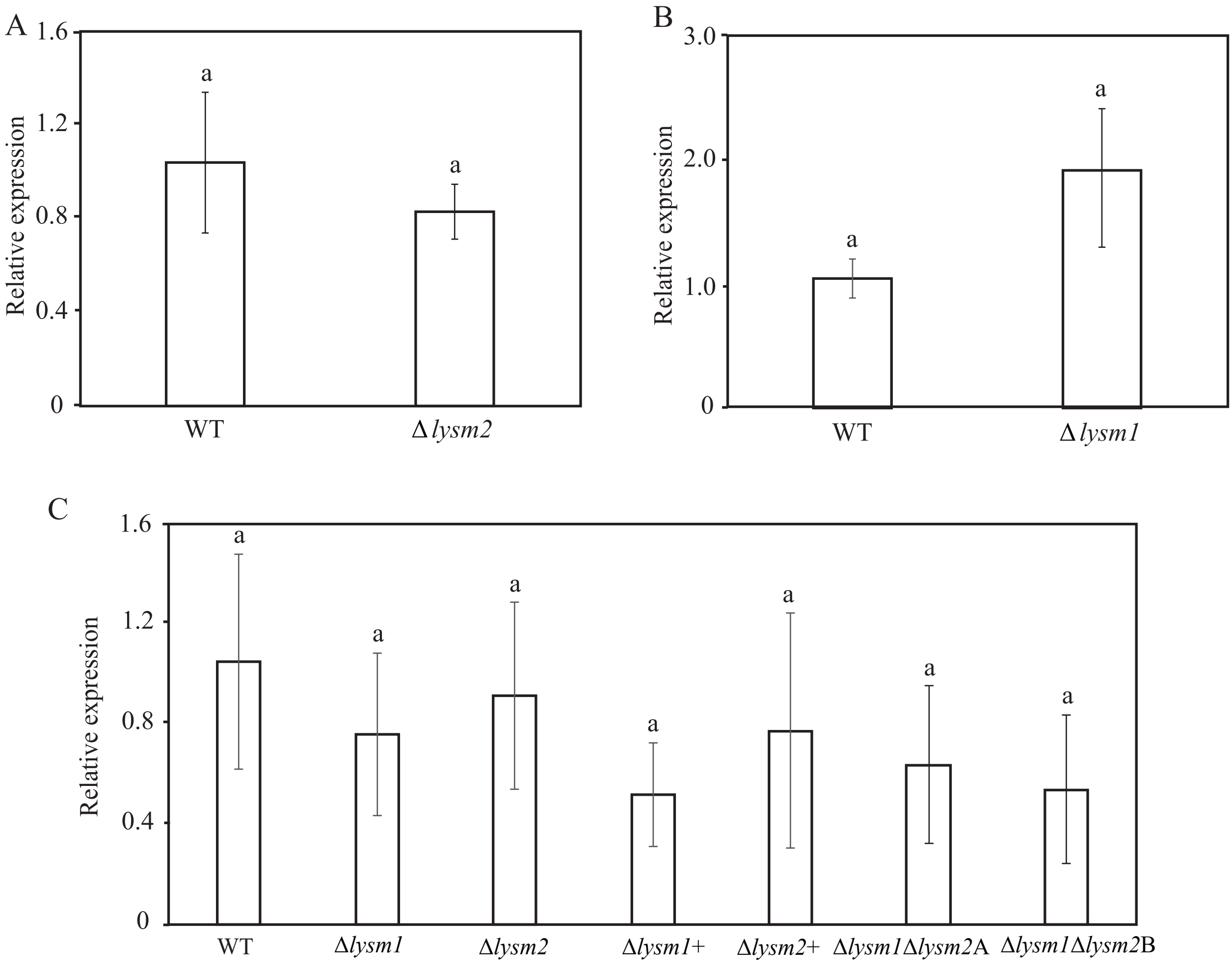

Figure S7: Expression analysis of *lysm1*, *lysm2* and *chiC2* in *C. rosea* WT and deletion strains (A) Expression analysis of *lysm1* in *C. rosea* WT and Δ*lysm2* deletion strains. (B) Expression analysis of *lysm2* in *C. rosea* WT and Δ*lysm1* deletion strain. (C) Expression analysis of *chiC2* in *C. rosea* WT, single deletion strains Δ*lysm1*and Δ*lysm2*, complemented strains Δ*lysm1*+ and Δ*lysm2*+ and double deletion strains Δ*lysm1*Δ*lysm2*A and Δ*lysm1*Δ*lysm2*B. Total RNA was extracted from the mycelia grown in SMS medium and used as template for cDNA synthesis. Expression level was normalized by β-tubulin expression using $2^{-\Delta\Delta Ct}$ method [66]. Error bar represent standard deviation based on four (*lysm1* and *lysm2*) or five (*chiC2*) biological replicates. Same letter indicates no statistically significant differences ($P \leq 0.05$) between treatments based on Student's *t* test or Fisher's exact test.
